# Phase 1 Trial of Malaria Transmission Blocking Vaccine Candidates Pfs25 and Pvs25 Formulated with Montanide ISA 51

**DOI:** 10.1371/journal.pone.0002636

**Published:** 2008-07-09

**Authors:** Yimin Wu, Ruth D. Ellis, Donna Shaffer, Erica Fontes, Elissa M. Malkin, Siddhartha Mahanty, Michael P. Fay, David Narum, Kelly Rausch, Aaron P. Miles, Joan Aebig, Andrew Orcutt, Olga Muratova, Guanhong Song, Lynn Lambert, Daming Zhu, Kazutoyo Miura, Carole Long, Allan Saul, Louis H. Miller, Anna P. Durbin

**Affiliations:** 1 Malaria Vaccine Development Branch, National Institute of Allergy and Infectious Diseases, Rockville, Maryland, United States of America; 2 Center for Immunization Research, Department of International Health, Johns Hopkins Bloomberg School of Public Health, Baltimore, Maryland, United States of America; 3 Biostatistics Research Branch, National Institute of Allergy and Infectious Diseases, Bethesda, Maryland, United States of America; Columbia University, United States of America

## Abstract

**Background:**

Pfs25 and Pvs25, surface proteins of mosquito stage of the malaria parasites *P. falciparum* and *P. vivax,* respectively, are leading candidates for vaccines preventing malaria transmission by mosquitoes. This single blinded, dose escalating, controlled Phase 1 study assessed the safety and immunogenicity of recombinant Pfs25 and Pvs25 formulated with Montanide ISA 51, a water-in-oil emulsion.

**Methodology/Principal Findings:**

The trial was conducted at The Johns Hopkins Center for Immunization Research, Washington DC, USA, between May 16, 2005–April 30, 2007. The trial was designed to enroll 72 healthy male and non-pregnant female volunteers into 1 group to receive adjuvant control and 6 groups to receive escalating doses of the vaccines. Due to unexpected reactogenicity, the vaccination was halted and only 36 volunteers were enrolled into 4 groups: 3 groups of 10 volunteers each were immunized with 5 µg of Pfs25/ISA 51, 5 µg of Pvs25/ISA 51, or 20 µg of Pvs25/ISA 51, respectively. A fourth group of 6 volunteers received adjuvant control (PBS/ISA 51). Frequent local reactogenicity was observed. Systemic adverse events included two cases of erythema nodosum considered to be probably related to the combination of the antigen and the adjuvant. Significant antibody responses were detected in volunteers who completed the lowest scheduled doses of Pfs25/ISA 51. Serum anti-Pfs25 levels correlated with transmission blocking activity.

**Conclusion/Significance:**

It is feasible to induce transmission blocking immunity in humans using the Pfs25/ISA 51 vaccine, but these vaccines are unexpectedly reactogenic for further development. This is the first report that the formulation is associated with systemic adverse events including erythema nodosum.

**Trial Registration:**

ClinicalTrials.gov NCT00295581

## Introduction

Despite decades of effort, malaria remains a major cause of morbidity and mortality in humans, causing 300 million clinical cases and over one million deaths annually [1]. Of the four species of malaria parasites that infect humans, *Plasmodium falciparum* is responsible for the majority of these deaths while *P. vivax* accounts for over 50% of all malarial infections outside Africa as well as 10% of those in Africa. Spreading drug resistance of the malaria parasite makes chemotherapy increasingly difficult. Alternative strategies to prevent the infection and disease spread are urgently needed.

Malaria is transmitted by *Anopheles* mosquitoes. Measures to block transmission have been integral to malaria control strategies. Use of insecticide-treated bed nets in endemic regions reduces the number of infectious bites thereby reducing morbidity and mortality [2]. Malaria transmission blocking vaccines, which target the sexual development of the parasite in the mosquito vector, could be another effective means to reduce malaria transmission. Overall, a sustained reduction in transmission, when used as a component of a control program, could contribute to sustained local elimination of the disease.

Pfs25 and Pvs25 proteins expressed on the surface of ookinetes in the mosquito stage of *P. falciparum* and *P. vivax*, respectively, are leading candidates for malaria transmission blocking vaccines. In animal studies, these antigens are able to generate strong transmission blocking activity [3]. In an early human trial volunteers were primed with NYVAC-Pf7, a viral vector expressing seven *P. falciparum* genes including the Pfs25 gene, followed by a boosting dose with a recombinant Pfs25 protein [4]. These volunteers developed some anti-Pfs25 antibodies that had marginal activity in blocking transmission. The first human vaccine trial targeting *P. vivax* malaria transmission tested a recombinant Pvs25 formulated with Alhydrogel. Antibodies that developed in some of these volunteers were able to significantly inhibit parasite development in mosquitoes [5], but the levels were too low for an effective vaccine.

In order to enhance the immunogenicity of Pfs25 and Pvs25, we selected Montanide ISA 51 to formulate the vaccines as water-in-oil emulsions. Montanide ISA 51 has been used as an experimental adjuvant for protein and peptide vaccines. Like Freund's Incomplete Adjuvant (FIA), it is based on mineral oil with a mannide mono-oleate emulsifier. In contrast to FIA, a highly purified emulsifier is used in Montanide ISA51, thus avoiding impurities that contributed to the toxicity of some batches of FIA. It also uses a different ratio of emulsifier to oil, resulting in a more consistent and controllable emulsion [6]–[8]. Experience in human trials indicated that Montanide ISA 51 was an effective immune enhancer and was generally well tolerated. Most reported adverse reactions were transient local reactions, including local swelling and pain with or without fever. In these studies, mild injection site nodules were reported that resolved within weeks without medical intervention [9]–[13].

In this paper we report the results of a Phase 1 trial designed to evaluate the safety and immunogenicity of the malaria transmission blocking vaccine candidates Pfs25/ISA 51 and Pvs25/ISA 51 in healthy US adults. This is the first report of erythema nodosum and leukemoid reactions associated with a combination of an antigen with the Montanide ISA 51 adjuvant.

## Methods

The protocol for this trial and supporting CONSORT checklist are available as supporting information; see Checklist S1 and Protocol S1.

### Participants

Participants were healthy males and non-pregnant females between 18 and 50 years old recruited from the metropolitan Washington DC area. Exclusion criteria included previous receipt of a malaria vaccine, splenectomy, known immunodeficiency, use of systemic corticosteroids or immunosuppressive drugs, recent receipt of a licensed vaccine or blood products, recent or simultaneous participation in another investigational drug or vaccine study, abnormal screening laboratories (complete blood count, aspartate aminotransferase, alanine aminotransferase, creatinine) or positive urine β-hCG, serologic evidence of hepatitis B or C infection, antibody to HIV, or any other clinically significant disease or condition which might confound the interpretation of study results. A prior history of malaria or recent travel to a malaria endemic area was not an exclusion criterion.

### Ethics

The study was conducted under a protocol reviewed and approved by the Committee on Human Research (the Johns Hopkins Bloomberg School of Public Health Institutional Review Board) and the Institutional Review Board of the National Institute of Allergy and Infectious Diseases. The study protocol was submitted to the U.S. Food and Drug Administration for review as part of Investigational New Drug application BB-IND #12163. The study was monitored for regulatory compliance and data quality assurance by the NIAID Regulatory Compliance and Human Subjects Protection Branch. Written informed consent was obtained from all volunteers prior to screening for eligibility for enrollment, in accordance with the Code of Federal Regulations Title 21, Part 50.

### Intervention: Study Agents

Recombinant proteins Pfs25 and Pvs25 were produced in the yeast expression systems utilizing *Pichia pastoris* and *Saccharomyces cerevisiae*, respectively [14]–[16]. A hexa-His tag was added to the C-terminus of the recombinant proteins to facilitate purification and characterization. Clinical grade Pfs25 and Pvs25 were produced at the Department of Biologics Research Pilot Bioproduction Facility, Walter Reed Army Institute of Research (WRAIR, Silver Spring MD), under current Good Manufacturing Practices (cGMP) conditions. Each protein was supplied in sterile solution, and underwent comprehensive quality control analyses to ensure purity, identity, and integrity.

The vaccines were formulated as previously described with slight modifications [17]. Briefly, the Pfs25 or Pvs25 at a concentration of 320 µg/mL in phosphate-buffered saline (PBS, 155 mM NaCl, 1 mMKH_2_PO_4_, 3 mM Na_2_HPO_3_) was aseptically emulsified with an equal volume of Montanide ISA 51 to give a final vaccine concentration of 160 µg/mL. The emulsion was achieved by homogenizing the mixture in a volume of 200 mL in a 400-mL vessel at room temperature for 6 min at 6000 rpm using an Omni Mixer-ES homogenizer (Omni International, Warrenton, VA). Three lots of clinical grade vaccines were prepared and vialed by the Pharmaceutical Development Section, The Clinical Center, National Institutes of Health: Pfs25/ISA 51, 160 µg/mL; Pvs25/ISA 51, 160 µg/mL; and an adjuvant control vaccine containing PBS/ISA 51, with volume weighted average droplet sizes of 0.816 µm, 0.803 µm, and 0.824 µm, respectively. Each vaccine lot underwent comprehensive quality control analyses to ensure general safety, purity, identity, integrity, and uniform water-in-oil droplet size. Stability of vaccines stored at 2–8°C was evaluated regularly using mouse immunogenicity tests and physical and biochemical assays to verify the vaccine safety, potency, uniformity, purity, and integrity until 4–10 months after the termination of the human immunizations (Table 1).

**Table 1:** Results of Stability Studies Performed on Pfs25/ISA 51, Pvs25/ISA 51, and PBS/ISA 51.

Tests^a^	Sept. 2004	Mar. 2005	Sept. 2005	May 2006	Sept 2006
	8 mo prior^b^	2 mo prior^b^		6 mo post^b^	10 Mo post^b^
**Endotoxin**	Pass	n.d.	Pass	n.d.	Pass
**Sterility**	Pass	n.d.	Pass	n.d.	Pass
**Potency** c	Pass	Pass	Pass	Pass	Pass
**Color and Appearance**	Pass	Pass	Pass	Pass	Pass
**Droplet Size**	Pass	Pass	Pass	Pass	Pass
**Identity by SDS-PAGE and Western Blot** c	Pass	Pass	Pass	Pass	Pass
**Integrity by SDS-PAGE** c	Pass	Pass	Pass	Pass	Pass
**Protein Content** c	Pass	Pass	Pass	Pass	Pass

a Except for endotoxin and sterility tests, all other tests were performed in comparison with a freshly prepared vaccine as a reference. “Pass” indicated results obtained from the stored vaccine were comparable to its reference.

b Time point relative to the vaccination period (May–October, 2005).

c These tests are only applied to Pfs25/ISA 51 and Pvs25/ISA 51 vaccines.

The 160 µg/mL Pfs25/ISA 51 and 160 µg/mL Pvs25/ISA 51 vaccines were diluted with the PBS/ISA 51 (the adjuvant control vaccine) to the final dose forms of 10 µg/mL or 40 µg/mL prior to immunizations. As a result of different degrees of dilution, these vaccines contained two different ratios of vaccine-containing vs. vaccine-free water droplets, namely ratios of 1∶15 and 1∶3 for the 10 µg/mL and 40 µg/mL formulations, respectively. The test and control vaccines were highly viscous and required vortexing prior to and after manipulation to ensure homogeneity.

### Intervention: Toxicology Study in Rabbits

To support the clinical use of the Pfs25/ISA 51 and Pvs25/ISA 51 vaccines, a toxicology study was conducted with New Zealand White rabbits in compliance with Good Laboratory Practice standards (GeneLogic, Gaithersburg, Maryland). Rabbits were selected as the model species because they produce immune responses to both vaccines, and they can be administered the full dose intended for humans by the intramuscular route. A 14-week study was performed using 84 rabbits in 4 groups. Groups 1, 2, and 3 (24 rabbits in each group, 12 males and 12 females) were each given 80 µg Pfs25/ISA 51 vaccine, 80 µg Pvs25/ISA 51 vaccine, or PBS/ISA 51 in a volume of 0.5 mL, respectively. The preclinical vaccines were prepared following the same procedures as for the clinical vaccines described above. The fourth group containing 6 male and 6 female rabbits was given PBS alone as a control. Although we planned to give human volunteers 2 vaccinations at a 4-month interval, the toxicology study design was more aggressive: each rabbit received 4 immunizations at 1-month intervals. Animals were observed during the course of the study for morbidity, mortality, general health and signs of toxicity, food consumption, body weight, clinical observations including skin and fur characteristics, eye and mucous membrane, respiratory, circulatory, autonomic and central nervous systems, somatomotor and behavior patterns, ophthalmologic examination, and dermal Draize observations. Hematology, blood chemistry, and immunological analyses were performed on days 4, 32, 60, 88 and 99 (approximately 4 days post-each immunization). Major tissues and organs were examined histologically on necropsy. Half of groups 1, 2 and 3 were sacrificed 3 days after the fourth immunization (day 88) for assessment of acute local reactogenicity. The remaining half of the three groups was sacrificed 14 days after the fourth immunization (day 99). The PBS control group was sacrificed 14 days after the fourth immunization.

### Intervention: Phase 1 Study Design

This was a partially blinded, dose escalating, controlled Phase 1 study in healthy US volunteers. The study was intended to enroll 10 volunteers in each of three dose levels (5, 20, and 80 µg per dose in 0.5 mL) for both vaccine candidates, and a total of 12 controls for a total of 72 volunteers. Investigators were blinded as to whether or not a volunteer received vaccine or the adjuvant control, but were aware of the dose of vaccine to be administered. Volunteers were randomly assigned to receive Pfs25/ISA 51, Pvs25/ISA 51, or PBS/ISA 51, and were sequentially assigned to higher dose groups as they were enrolled. Vaccinations were staggered within each dose cohort such that 4 volunteers were vaccinated a minimum of three weeks prior to the remaining 8 volunteers. A minimum interval of three weeks was required between completion of vaccination of the first dose cohort and start of vaccination of the next dose cohort. In addition, approval by the Safety Monitoring Committee (SMC) was required prior to escalation to a higher dose for each vaccine. Volunteers were scheduled to receive two immunizations 4 months apart. Vaccinations were given by IM injection in the deltoid muscle, with the successive vaccination given in the alternate arm.

### Objectives

The primary objective of this Phase 1 trial was to assess the safety, reactogenicity, and immunogenicity of the transmission blocking vaccine candidates Pfs25 and Pvs25, both adjuvanted with Montanide ISA 51. Secondary objectives were to assess the duration of specific antibody response over an 18 month period and to assess the effect of a second booster dose on antibody levels. Tertiary objectives were to measure the ability of vaccine-induced antibody to inhibit oocyst development in a mosquito membrane feeding assay, to determine the relationship between antibody levels and degree of transmission blocking, and to establish human standards for immune assays to be used for further development of transmission blocking vaccines.

### Outcomes: Safety and Tolerability

Safety and tolerability outcomes were the frequency of immediate, systemic, and local adverse events, by severity and relationship to vaccine. Following each vaccination, volunteers were observed for 30 minutes for immediate adverse reactions. The follow-up visits were scheduled for days 1, 3, 7, 14, and 21 for evidence of local and systemic reactogenicity. Volunteers were seen again at study days 30, 60, and 90 following vaccination for evaluation of any acute complaints, local reactogenicity, and assessment of immune status. Volunteers returned on study day 120 for a second vaccination and were followed on the same schedule as described above, with the exception of the study day 90 visit. Volunteers were seen 5 months, 8 months and 12 months following second vaccination for physical evaluation and immunological assessment. Local adverse events included erythema, induration, swelling, and tenderness at the site of injection. Solicited systemic adverse events included fever (oral temperature≥37.5°C), headache, nausea, malaise, myalgia, and arthralgia. Volunteers recorded local and systemic events daily, as well as their oral temperature three times daily, on diary cards for twenty days following each vaccination. An abbreviated history and physical examination was performed at each follow-up visit. All new or abnormal signs and symptoms were considered as adverse events. Each adverse event was graded for severity and assigned causality relative to the study vaccine. Severity was graded as either absent/none (Grade 0), mild (Grade 1, easily tolerated), moderate (Grade 2, interfered with activities of daily living), or severe (Grade 3, prevented activities of daily living). Erythema, induration, and swelling at the injection site were graded as follows: 0 = absent, 1 = 0–20 mm, 2 = 20–50 mm, 3 = >50 mm. Fever (oral) was graded as 0 = <37.5°C, 1 = 37.5–38°C, 2 = 38–39°C, and 3 = >39°C. A complete blood count and white blood cell differential, as well as serum creatinine and aspartate amino transferase (AST) concentrations were performed immediately prior to each vaccination and on days 3, 14, and 60 after each vaccination. A complete blood count was also obtained on day 7 following vaccination. Serious adverse events (SAEs) were defined as any adverse event resulting in death, life threatening, requiring hospitalization, resulting in disability or incapacity or congenital anomaly or birth defect, or any other event which required intervention to prevent such outcomes.

### Outcomes: Immunogenicity

Anti-Pfs25 and anti-Pvs25 IgG levels induced by the vaccines were evaluated by a standard enzyme-linked immunosorbent assay (ELISA) to measure serum antibodies to Pfs25 and Pvs25 proteins [18]. Briefly, ELISA plates were coated with Pfs25 or Pvs25. Sera collected from volunteers were tested against a set of serially diluted reference standard serum. The anti-Pvs25 reference standard serum was generated as described previously [19], [20]. The anti-Pfs25 reference standard serum was generated from one volunteer who developed significant antibody responses after the second immunization. These reference standards were assigned ELISA unit values equal to the reciprocal of the dilution giving an OD_405nm_ of 1. Absorbance of the set of serially diluted reference standards was fitted to a 4-parameter hyperbolic function to generate a standard curve. Using this standard curve, the absorbance of an individual test serum was converted to an antibody unit value.

### Outcomes: Transmission Blocking Activity

Transmission blocking activity (TBA) of the sera was tested by an ex vivo membrane feeding assay as described previously[21] with following modifications: test sera were heat-inactivated and diluted with a naïve human serum pool to a desired concentration. Purified IgGs were buffer-exchanged and concentration-adjusted with PBS prior to dilution with the naïve human serum pool. An in vitro gametocyte culture of *P. falciparum* (NF54 line) was evaluated for percentage of Stage V gametocytes (>0.5%) and the vitality of exflagellation centers observed at 400× magnification. The culture pellet was diluted with normal O+ RBC (Interstate Blood Bank, Memphis, Tennessee) and normal heat-inactivated O+ human serum pool (Interstate Blood Bank, Memphis, Tennessee) to achieve 0.15%±0.05% concentration of Stage V gametocytes and haematocrit of 50%. This infected blood mixture was kept at 37°C, and aliquoted into 200 µl prior to the feed. One 200 µl -aliquot of “infected blood” was mixed with 60 µl of the test serum or antibodies, and immediately fed to 3–8 days old *Anopheles stephensi* (Nijmegen strain) mosquitoes, starved for 24–30 hours, through a membrane feeding apparatus using a thin stretched parafilm membrane. Mosquitoes were kept for 7–8 days after the feed at 26°C and high humidity conditions to allow parasites to develop into oocysts. Infectivity was measured by dissecting at least 20 mosquitoes per sample, staining the midguts with 0.05% mercurochrome solution in water for at least 20 min, and counting the number of oocysts in each midgut. The feeding experiment was not analyzed unless the average oocyst count in mosquitoes fed with naïve human serum pool was more than four. Percent inhibition of oocyst development per mosquito was determined by the formula:
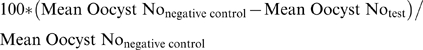
where the negative control feed used pre-vaccination sera from the same volunteer. All samples or diluted samples were tested in replica.

### Sample size

A group size of 10 volunteers per dose was chosen based on the distribution of antibody responses from previous clinical trials [22], [23], and would permit detection of at least a five-fold difference in antibody concentration between dose groups using a Mann-Whitney test, assuming a level of significance of 0.05 and a power of 0.80. This sample size also gave 0.80 probability for detecting one or more adverse events that occurred with a probability of 0.15 per volunteer.

### Randomization—Sequence generation and Implementation

Volunteers in each cohort were randomly assigned to receive vaccine or the adjuvant control. The study pharmacist determined all treatment assignments by the use of a random number generator prior to enrollment of any volunteers. Clinical staff enrolled participants sequentially into each vaccine cohort. Each cohort was enrolled separately for safety reasons, as stated above.

### Randomization—Allocation concealment

The treatment assignment log was kept in a locked file cabinet to which only pharmacy staff had access. Each syringe was labeled with the volunteer number and expiration time of the vaccine. The syringe with the test article cohort was labeled in such a way that the identity of the test article (vaccine or control) was not apparent. Both vaccine and control were opaque white in appearance and therefore indistinguishable.

### Statistical methods

The study was terminated before completion of the scheduled vaccinations. Due to the small numbers of subjects enrolled in the study, statistical analysis regarding the frequency of adverse events and antibody responses was not performed as planned.

The TBA is expressed as a percentage of inhibition of parasite development in mosquitoes. Hill Plot [24] was used to model the relationship between TBA and ELISA, as defined by formula:
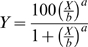
where Y is the percent reduction of oocyst number, *X* is the ELISA units in the test sample, *a* is the Hill coefficient, and *b* is ELISA unit value that would result in 50% of reduction of the oocyst number (IC_50_), using *R* (Version 2.6.0) [25]. The fit was weighted by least squares with weights equal to the inverse of the variance, which was estimated by the delta method [26].

## Results

### Participant Flow/Recruitment

**Figure 1:**
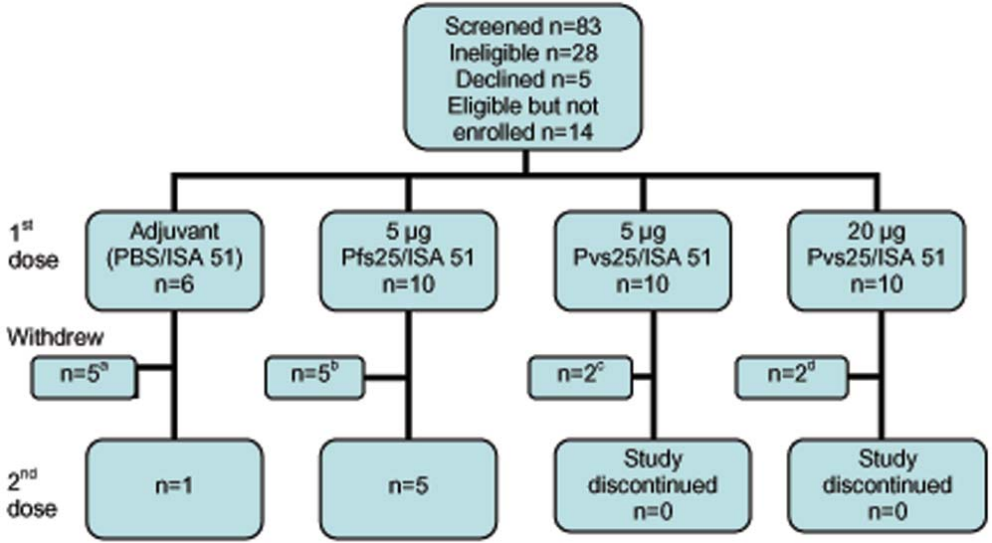
Participant Recruitment and Flow. ^a^ One volunteer withdrew consent; one lost to follow up; study discontinued prior to second vaccination of the 3 volunteers. ^b^ One withdrew consent; two were withdrawn due to local reactions (severe swelling and induration in one volunteer and severe swelling, induration, and pain in the other); two were ineligible due to previously undisclosed exclusion criteria (concurrent participation in another investigational drug trial and mental illness). ^c^ Two were withdrawn due to local reactions (severe swelling and induration in one volunteer and severe swelling, induration, tenderness, and pain in the other). ^d^ Two were withdrawn due to systemic adverse events (erythema nodosum).

Thirty-six volunteers were enrolled in the study from May through October 2005. Participant recruitment and flow are shown in Figure 1. The study was stopped prior to completion of enrollment due to unacceptable reactogenicity (see below). The mean age of the volunteers at enrollment was 32.4 years (range 19–49), with no significant difference in the ages of volunteers in each dose cohort (data not shown). Forty-seven percent (47%) of volunteers identified themselves as Caucasian; 39% as African American; six percent as Hispanic; 6% as Asian; and 1 volunteer identified as “multi-racial”.

Ten volunteers in each of the 5 µg Pvs25/ISA 51, 20 µg Pvs25/ISA 51, and 5 µg Pfs25/ISA 51 dose groups were enrolled and received their first vaccinations. In the 5 µg Pfs25/ISA 51 cohort, 5 of the volunteers received a second dose; 2 did not receive the second vaccine because of a serious local reaction following the first vaccination; 1 withdrew his consent prior to the second vaccination; and 2 volunteers were ineligible for second vaccination (1 was out of the protocol-defined window for vaccination and the other had a medical condition unrelated to the first vaccination which excluded her from further vaccination). Six volunteers were randomized to receive adjuvant control vaccine (PBS/ISA 51); 5 of those received one dose and 1 received two doses. Because further vaccinations were stopped at this point due to unacceptable reactogenicity, as discussed below, volunteers in the other dose cohorts did not receive a second vaccination. Volunteers who received both doses of vaccine and the volunteers who developed erythema nodosum (see below) were followed for a total of 18 months. Volunteers who received only one vaccination were followed for a total duration of six months.

### Outcomes and Estimation

#### Results of the Toxicology Study in Rabbits

Significant specific antibody responses were induced by the repeated immunizations of the test vaccines. No vaccine-related changes were observed in mortality, clinical observations, body weights, organ weights, food consumption, gross and clinical pathology, ophthalmology, or gross necropsy observations. Minimal to mild edema and/or erythema at the injection sites were observed in all rabbit groups, including the group receiving PBS only, with relatively similar incidence and severity. Histopathology showed inflammation at the injection sites in all groups receiving Montanide ISA 51 formulations, including Pfs25/ISA 51, Pvs25/ISA 51, and PBS/ISA 51. The inflammation characteristics among the groups varied slightly, and the presence of Pfs25 (the Pfs25/ISA 51 group) and Pvs25 (the Pvs25/ISA 51 group) did not significantly alter this profile, indicating the inflammation was due to the adjuvant Montanide ISA 51.

#### Safety and Tolerability Data of the Phase 1 Trial

*Local adverse events*

In the groups receiving antigen with ISA 51, 6 volunteers experienced severe local reaction, 4 experienced moderate local reaction, and 14 experienced mild reaction (maximum severity for each). Four of six volunteers who received the control vaccine (PBS/ISA 51) complained of mild injection site pain lasting up to 4 days and two recipients reported mild erythema for one day. Local adverse events are presented in Table 2. Injection site induration began 13 to 21 days after vaccination and, in all but one volunteer, resolved within eight weeks. Induration in this volunteer slowly resolved over the course of approximately six months. The induration observed in volunteers receiving 5 µg dose of Pfs25/ISA 51 was much more firm than that observed in recipients of either dose of Pvs25/ISA 51. With the exception of mild pain at the injection site, the incidence of local adverse events did not increase in volunteers who received a second dose of Pfs25/ISA 51 or in volunteers who received the higher 20 µg dose of Pvs25/ISA 51. One volunteer in the 20 µg Pvs25/ISA 51 group developed a sterile abscess at the injection site, started on day 23 and lasted 19 days and drained spontaneously; this volunteer also had erythema nodosum as described below.

**Table 2:** Number of volunteers in each vaccination group experiencing the indicated solicited local adverse event.

Vaccination Group	N^a^	Pain Grade			Tenderness Grade			Induration Grade^b^			Swelling Grade^b^			Erythema Grade^c^
		1	2	3	1	2	3	1	2	3	1	2	3	1
**5 µg Pfs25/ISA51 Dose 1**	10	2	2	1	1	0	0	0	0	2	1	1	3	3
**5 µg Pfs25/ISA51 Dose 2**	5	4	0	0	2	0	0	0	0	0	0	1	0	1
**5 µg Pvs25/ISA51 Dose 1**	10	5	1	1	3	2	1	0	2	2	0	0	2	0
**20 µg Pvs25/ISA51 Dose 1**	10	6	2	0	2	1	0	0	0	1	1	1	0	2
**PBS/ISA51**	6	4	0	0	1	0	0	0	0	0	0	0	0	2

a Number of volunteers in the vaccination group.

b Grade 1 = 0–20 mm, Grade 2 = 20–50 mm, Grade 3 = >50 mm.

c All erythema was Grade 1.

*Systemic and laboratory adverse events*

Related (possibly, probably, or definitely) solicited systemic adverse events are shown in Table 3. Systemic adverse events included mild to severe arthralgia, headache, malaise, myalgia, and nausea. Following first vaccination with 5 µg Pfs25/ISA 51, mild arthralgia and headache lasting 1–2 days were reported by one volunteer. Following second vaccination, one subject reported headache, malaise, and nausea for 1–2 days. One volunteer had Grade 1 leukopenia on two instances: 57 days following first vaccination and 14 days following second vaccination. The first episode of leukopenia resolved after ninety days and the second episode resolved within 7 days of onset; only the second was judged to be possibly related to vaccination. This subject also developed mild ketonuria thought to be possibly related to vaccination. One volunteer developed a severe leukemoid reaction, described below.

**Table 3:** Number of volunteers in each vaccination group experiencing the indicated solicited adverse event (possibly, probably, or definitely related to vaccination).

Vaccination Group	N^a^	Headache Grade			Arthralgia Grade			Myalgia Grade			Malaise Grade			Nausea Grade^b^	Leukopenia Grade^b^
		1	2	3	1	2	3	1	2	3	1	2	3	1	1
**5 µg Pfs25/ISA51 Dose 1**	10	1	0	0	1	0	0	0	0	0	0	0	0	0	0
**5 µg Pfs25/ISA51 Dose 2**	5	1	0	0	0	0	0	0	0	0	0	1	0	1	1
**5 µg Pvs25/ISA51 Dose 1**	10	1	0	0	0	0	0	0	0	0	0	0	0	0	1
**20 µg Pvs25/ISA51 Dose 1**	10	0	0	1	0	1	1	0	1	1	0	0	1	1	0
**PBS/ISA51**	6	1	0	0	0	0	0	0	0	0	2	0	0	1	1

a Number of volunteers in the vaccination group.

b All nausea and leukopenia were Grade 1.

Systemic adverse events after one dose of 5 µg Pvs25/ISA 51 included mild headache in one volunteer. Three volunteers developed grade 1 leukopenia, however the onset of leukopenia was greater than 120 days after first vaccination in two of the three volunteers and these were judged to be unlikely to be related to vaccination. Systemic events after one dose of 20 µg Pvs25/ISA 51 included two cases of erythema nodosum with associated headache, arthralgia, and myalgia, and a leukemoid reaction (described below).

*Leukemoid reactions*

Two volunteers developed leukemoid reactions during the course of this study. One volunteer in the 5 µg Pfs25/ISA 51 group developed a leukemoid reaction 14 days after receiving his second dose of vaccine, with a total white blood cell (WBC) count of 35,700 cells/µl, and an absolute neutrophil count 27,489 cells/µL with 2% bands (left shift). The volunteer was clinically well, and reported that he had donated blood one day before at his workplace requiring three attempts at phlebotomy. The CBC was repeated one day later and WBC count was 14,500/µL. Blood cultures were also obtained and were negative; C-reactive protein (CRP) level and erythrocyte sedimentation rate were normal. The WBC count returned to baseline within one week and remained within normal limits. The volunteer remained otherwise well for the duration of the study. The leukemoid reaction was graded as possibly related to vaccination.

One volunteer in the 20 µg Pvs25/ISA 51 group was seen for regularly scheduled study follow up 120 days after receiving vaccination. She was clinically well at that time but her WBC count was 34,600 cells/µL with a left shift. The volunteer did not return to the clinic for repeat lab testing and evaluation until four weeks later, despite numerous requests. Her WBC count was repeated at that time and was normal. It remained normal on subsequent visits. This reaction was also graded as possibly related to vaccination.

*Erythema nodosum*

Two volunteers who received one dose of 20 µg Pvs25/ISA 51 developed erythema nodosum, both at 18 days after vaccination. One volunteer was a 25 year-old Asian female; the other was a 26 year old Caucasian female; both were taking oral contraceptives. Symptoms in the 25 year old were graded as moderate and included painful nodules on the lower legs, and erythematous macules on the arms and legs. The other volunteer had symptoms graded as severe with fever, headache, arthralgia, and conjunctivitis in addition to painful nodules on her anterior lower legs. A transient elevation of CRP occurred in both volunteers: a CRP level of 75 mg/L (upper limit of normal is < 8 mg/L) was seen in the volunteer with the severe reaction while the other volunteer had only a mild elevation of CRP with a value of 8 mg/L. An elevated level of complement (CH50) was also found in the volunteer with the moderate reaction. A dermatology consult was obtained and erythema nodosum-like syndrome was diagnosed in both volunteers, with skin biopsy results for the patient with the severe reaction showing non-specific panniculitis consistent with erythema nodosum. The other volunteer did not undergo a skin biopsy. The severe reaction resolved at 38 days, and the moderate reaction resolved at 12 days. Both were graded as probably related to vaccination.

This study was closed to enrollment and vaccinations were stopped due to the erythema nodosum reactions.

#### Immunogenicity Data

Pvs25/ISA 51 group: Sixteen of the 20 volunteers, including one with a leukemoid reaction and one of the two with grade 3 induration, failed to develop detectable antibodies against Pvs25 (i.e. antibody levels were lower than 25 ELISA units, the background level) at any time during the follow-up period. The other volunteer with grade 3 induration developed measurable antibodies with a peak level of 96 units on day 120. Antibodies were first detectable at day 60, about 3 weeks following resolution of the induration. Both volunteers with erythema nodosum had detectable antibody levels starting at day 30. The volunteer with a severe reaction had a peak antibody level of 219 ELISA units on day 60, whereas the volunteer with a moderate reaction had a peak level of 583 units on day 150. The fourth volunteer developed antibody levels of 34 units on day 60 and 84 units on day 120, and had no significant adverse reactions.

Pfs25/ISA 51 group: The antibody responses against Pfs25 are detailed in Table 4. Four of 10 volunteers, including the one that developed a leukemoid reaction (Volunteer “C”), had no detectable antibodies against Pfs25 (i.e. <25 ELISA units) by day 120 following the first vaccination. Of the 2 volunteers that developed grade 3 induration, one (Volunteer “H”) had a minimal antibody level of 30 ELISA units on day 90, 30 days after the induration resolved. The other (Volunteer “G”) had 132 ELISA units on day 60. The antibody levels declined over time, along with the induration which resolved by Day 370. These 2 volunteers did not receive the second vaccination.

**Table 4:** Anti-Pfs25 Antibody Levels Following Vaccination of 5 µg Pfs25/ISA 51^a^.

Volunteer	Anti-Pfs25 ELISA Units on Study Days^c^											
	0	14	30	60	90	120	134	150	180	270	360	556
**A**	<25	<25	<25	<25	<25	<25	932	1527	672	167	95	52
**B**	<25	<25	<25	<25	<25	<25	263	631	362	116	64	40
**C**	<25	<25	<25	30	28	29	469	694	514	168	59	34
**D**	<25	<25	<25	<25	<25	36	1688	6329	7322	3657	1653	1022
**E**	<25	<25	<25	<25	<25	<25	347	665	745	204	108	n.d.
**F** b	<25	<25	<25	<25	<25	n.d.	n.d.	n.d.	<25	n.d.	n.d.	n.d.
**G** b	<25	<25	<25	132	123	100	n.d.	98	87	53	39	n.d.
**H** b	<25	<25	<25	<25	30	41	n.d.	37	39	36	n.d.	n.d.
**I** b	<25	<25	<25	<25	<25	<25	n.d.	n.d.	<25	n.d.	n.d.	n.d.
**J** b	<25	<25	<25	<25	n.d.	<25	<25	n.d.	<25	n.d.	n.d.	n.d.

a The volunteers were scheduled to receive 2 vaccinations on days 0 and 120.

b These volunteers did not receive the second vaccination.

c <25, below the assay detection limit; n.d., not tested because samples were not available.

All 5 volunteers (Volunteers “A” through “E”) receiving a second dose of 5 µg Pfs25/ISA 51 developed substantial antibody levels against Pfs25 following the second vaccination (Table 4). The antibody levels reached a peak 30–60 days after the second vaccination and the geometric mean of the peak of this group was 1295 ELISA units. The antibody slowly decayed, but all 5 volunteers maintained detectable antibody levels 1 year after vaccination.

#### Transmission Blocking Activity of the Antisera

**Figure 2:**
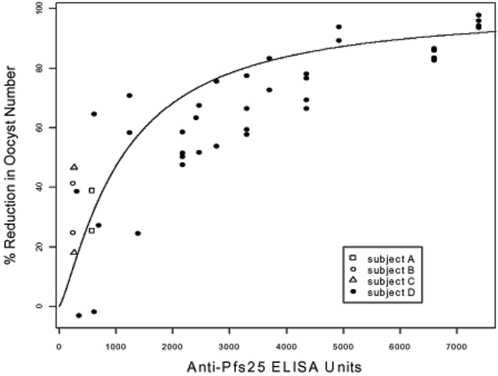
Correlation of anti-Pfs25H antibody units with transmission blocking activity measured as percent reduction in oocysts per mosquito. Sera or purified IgGs were tested in replica in various dilution. The non-linear equation: 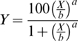 was used to fit the data, where variables *Y* and *X* are the percent reduction of oocyst number, and the ELISA units, respectively, the curve parameters were calculated as *a*  = 1.27 (95% CI 0.96–1.68) and *b* = 1093 (95% CI 682–1565).

Antisera from 4 of the 5 volunteers who completed 2 scheduled doses of 5 µg Pfs25/ISA 51 were tested for their ability to block transmission. In ex vivo membrane feeding assays, one antiserum that contained 7322 ELISA units resulted in reduction of the parasite in mosquitoes by >90%. The transmission blocking activity decreased when the serum was diluted in the assay. Three antisera had low to moderate antibody titers of 630 to 1520 ELISA units. These sera gave low to moderate transmission blocking activity (18–47% reduction in oocyst number). Figure 2 shows the relationship between antibody titer and transmission blocking activity in the sera. From the correlation between antibody and blocking, 1093 units (95% Confidence Interval, 683–1565 units) of antibody are required to reach a 50% reduction in oocyst density.

## Discussion

### Interpretation

This study involved the first in-human use of two novel antigen/adjuvant combinations. A previous clinical trial of one of these antigens, the same Pvs25 used in this study but adsorbed to Alhydrogel®, showed minimal reactogenicity, with injection site tenderness as the most commonly observed adverse event. However, the immunogenicity was poor with this formulation [27]. Two previous human trials with a similar recombinant Pfs25 antigen, one in which the antigen was adsorbed to Alhydrogel® and one where a prime/boost strategy was used, also showed an adverse event pattern limited to local reactogenicity, although one volunteer had an atypical contralateral hypersensitivity reaction thought to be due to free antigen dissociated from the alum [4], [28]. Due to Pfs25's poor immunogenicity and inadequate binding to Alhydrogel, a different adjuvant platform using Montanide ISA 51 was explored for its further clinical development

Montanide ISA 51 has been used as an adjuvant with investigational agents in a large number of clinical trials, with the most common adverse reactions being local injection site pain or swelling, although some studies have reported injection site nodules and sterile abscesses, [29]–[31]. Other water-in-oil formulations (e.g. FIAt and Montanide ISA 720) have also occasionally caused moderate to severe local reactions, including sterile abscesses. This class of adjuvant has thus far been free of reports of severe vaccine associated-systemic reactogenicity. Since the frequency of the local reactions varies greatly according to antigen and dose, it is difficult to predict if a new antigen will give rise to an unacceptable frequency of reactogenicity. In some situations, a relatively high incidence of moderate or severe local injection site reactions may be an acceptable safety risk in a vaccine with a high benefit potential, such as with malaria vaccines [32]. Therefore it was considered acceptable to proceed with a Phase 1 study with staggered vaccinations, designed to allow safety assessment prior to the next dose cohort and minimize the number of severe adverse events.

Despite the lack of issues identified in preclinical studies including a toxicology study conducted in rabbits, both Pfs25/ISA 51 and Pvs25/ISA 51 proved to be quite reactogenic in humans. Two types of local reactogenicity were observed: local tenderness and swelling occurring within a few days of vaccination, and induration that occurred approximately 2 weeks after vaccination. In this study development of induration did not seem to be associated with higher antibody response whereas in previous studies, indurations tended to be associated with the subjects with the highest antibody responses [33].

Although indurations and other local reactogenicity were of concern, these were not regarded as severe enough to stop the trial. The trigger to cease vaccination was development of erythema nodosum like symptoms in 2 of 10 volunteers who received 20 µg Pvs25/ISA 51. Erythema nodosum is an inflammatory panniculitis, commonly associated with underlying systemic disease or infections such as sarcoidosis or tuberculosis. It is more common in women and is also associated with the use of oral contraceptives [34]. Erythema nodosum has been reported in association with both plasma derived and recombinant hepatitis B vaccines, and with typhoid vaccination [35]–[39]. Given the parallel timing and presentations of these two reactions, the likelihood of a causal relationship to vaccination is high. The two leukemoid reactions were less similar in that they occurred in volunteers who received different vaccines, and one occurred 14 days after vaccination and one occurred 120 days after vaccination. However, given the absence of any other plausible explanation for these events a causal relationship is possible, and perhaps likely for the reaction which occurred 14 days after vaccination.

It is unlikely that observed systemic adverse events were associated with the antigen alone. Three cases of systemic reactions (2 erythema nodosum cases and 1 transient leukemoid case) occurred in cohorts receiving Pvs25/ISA 51 vaccine. The same production lot of Pvs25 was used to produce the Pvs25/Alhydrogel vaccine that was well tolerated in humans [40]. Both stored Pfs25/ISA 51 and Pvs25/ISA 51 vaccines met specified criteria in routine stability and potency studies (Table 1). Combining the evidence from the literature and our observations in this trial, it seems likely that the erythema nodosum reactions and possibly the transient leukemoid reactions were related to the specific antigen/adjuvant combinations. These reactions were not predicted by the toxicity study conducted in rabbits, nor have they been seen in other trials in mice, *Aotus* monkeys and rhesus monkeys.

Although the trial was stopped due to adverse events, the data from volunteers receiving 2 doses of 5 µg Pfs25/ISA 51 clearly show that it is possible to induce antibody in humans that can effectively block parasite development in mosquitoes. The data also enabled an estimate of antibody levels (1093 with 95% CI 683–1565 ELISA units) required to give 50% blockage of oocyst development. Using this figure and results from recent modeling of the impact of mosquito stage transmission blocking vaccines [41], the average antibody levels (geometric mean of 1295 units) in this dose group would correspond to a 1.5 fold decrease in inoculation rates if 90% of the gametocyte carriers in a village were vaccinated, even though 5 µg was the lowest dose group in the study.

### Generalization

In addition to local reactogenicity, this study observed for the first time an association between systemic adverse events and a specific antigen-Montanide ISA 51 formulation. These results highlight the need for careful trial design in Phase 1 studies with novel antigen/adjuvant combinations [42]. The induction of transmission blocking antibodies in volunteers who received the lowest scheduled doses of Pfs25/ISA 51 demonstrates the feasibility of this approach. However, the severity and duration of the local reactions seen in this study, combined with the observed systemic reactions, make further progression of the Montanide ISA 51 formulations unlikely. Current efforts are focused on the development of a well tolerated formulation capable of inducing strong immune responses.

## Supporting Information

Protocol S1 Trial Protocol.
(0.85 MB DOC)

Checklist S1 Consort Checklist.
(0.06 MB DOC)
