# Supplementary material for: Phase 1 Trial of Malaria Transmission Blocking Vaccine Candidates Pfs25 and Pvs25 Formulated with Montanide ISA 51
Source: PLoS One. 2008 Jul 9;3(7):e2636. doi: 10.1371/journal.pone.0002636 (PMC2440546; doi:10.1371/journal.pone.0002636)
Supplement: Protocol S1 — Trial Protocol. (0.85 MB DOC) [file pone.0002636.s001.doc]

**Phase 1 Study of the Safety and Immunogenicity of PpPfs25/ISA51 and ScPvs25/ISA51: Transmission Blocking Vaccines for *Plasmodium falciparum* and *Plasmodium vivax* Malaria**

Version: Version 4.0 (August 20, 2007)

NIAID Protocol Number: 05-I-0118

CHR Protocol Number: H.22.04.12.23.A1

FDA IND Number: BB-IND-12163

Multi-institution: No

Project Assurance: FWA #00000287

Proposed start date: January 2005

Proposed end date: May 2007

Sponsored by:

Regulatory Compliance and Human Subjects Protection Branch (RCHSPB)

Office of Clinical Research (OCR), Office of the Director (OD)

National Institute of Allergy and Infectious Diseases (NIAID)

National Institutes of Health (NIH)

Version 4.0: August 20, 2007

Previous Versions

Version 3.7: October 6, 2007

Version 3.6: January 13, 2006

Version 3.5: December 19, 2005

Version 3.4: November 7, 2005

Version 3.2: June 13, 2005

The information contained herein is the property of the National Institute of Allergy and Infectious Diseases and may not be reproduced, published or disclosed to others without the written authorization of the National Institute of Allergy and Infectious Diseases.

Team Roster

**Principal Investigator:** Anna Durbin, MD

Center for Immunization Research

Johns Hopkins School of Public Health

624 N. Broadway

Baltimore, MD 21205

(410) 955-1622

**Co-Investigators:**

Center for Immunization Research Clayton Harro, MD

Donna Shaffer, MS, RNCS, FNP

**Scientific Investigators:**

Malaria Vaccine Development Branch Yimin Wu, PhD

(MVDB) Louis Miller, MD

Carole Long, PhD

Siddhartha Mahanty, MD (Associate Investigator)

National Institute of Allergy and Infectious
 Diseases (NIAID), National Institutes of Health (NIH)

5640 Fishers Lane, Twinbrook I

Rockville, MD 20852

(301) 435-4305

**IND Sponsor Representative:** John Tierney, RN

RCHSPB, OCR, OD

NIAID, NIH

6700-B Rockledge Drive, MSC 7609

Bethesda, MD 20892-7609

(301) 451-5136

**Protocol Statistician:** Michael Fay, PhD

Biostatistics Research Branch

NIAID/NIH

6700A Rockledge Drive, Room 5133

Bethesda, MD 20892

(301) 451-5124

**Participating Sites**

**Clinical Trial Site:** Center for Immunization Research

Johns Hopkins School of Public Health

2112 F St, NW

Washington, DC 20037

202-223-6564

**Participating Laboratories**

**Immunology Laboratory:** **Malaria Vaccine Development Branch**

NIAID, NIH

5460 Fishers Lane, Twinbrook I

Rockville, MD 20852

(301) 435-3405

**Armed Force Research Institute of Medical Sciences**,

315/6 Rajvithi,

Bangkok, 10400

Thailand

Tel: 02-644-6733

**Clinical Laboratory:** **Quest Diagnostics**

1901 Sulphur Springs Road

Baltimore, MD 21227

(800) 368-2576

Apheresis Site: Department of Transfusion Medicine

Building 10 1C710, Clinical Center

National Institutes of Health

9000 Rockville Pike

Bethesda, Maryland 20892

(301) 496-1430

# Table of Contents

[1.0 Introduction 8](#__RefHeading___Toc147556288)

[1.1 Background 8](#__RefHeading___Toc147556289)

[1.2 Transmission Blocking Vaccines 8](#__RefHeading___Toc147556290)

[1.3 Vaccine Description 8](#__RefHeading___Toc147556291)

[1.3.1 Drug Substances 8](#__RefHeading___Toc147556292)

[1.3.2 Montanide® ISA51 9](#__RefHeading___Toc147556293)

[1.3.3 Vaccine Formulation 9](#__RefHeading___Toc147556294)

[1.4 Rationale 9](#__RefHeading___Toc147556295)

[1.4.1 Preclinical Experience 9](#__RefHeading___Toc147556296)

[1.4.2 Clinical Experience with PpPfs25H/ISA51 and ScPvs25H/ISA51 10](#__RefHeading___Toc147556297)

[1.4.3 Clinical Experience with Montanide® ISA51 and Related Adjuvants 10](#__RefHeading___Toc147556298)

[1.5 Clinical Development Plan 11](#__RefHeading___Toc147556299)

[1.5.1 Participation of Children 12](#__RefHeading___Toc147556300)

[2.0 Objectives 12](#__RefHeading___Toc147556301)

[2.1 Primary Objective 12](#__RefHeading___Toc147556302)

[2.2 Secondary Objectives 12](#__RefHeading___Toc147556303)

[2.3 Tertiary Objectives 12](#__RefHeading___Toc147556304)

[3.0 Study Design 12](#__RefHeading___Toc147556305)

[3.1 Overall Design 12](#__RefHeading___Toc147556306)

[3.2 Sample Size and Estimated Duration of Study 15](#__RefHeading___Toc147556307)

[3.3 Cohort Allocation 15](#__RefHeading___Toc147556308)

[4.0 Selection and Enrollment of Volunteers 15](#__RefHeading___Toc147556309)

[4.1 Inclusion Criteria 15](#__RefHeading___Toc147556310)

[4.2 Exclusion Criteria 15](#__RefHeading___Toc147556311)

[4.3 Treatments That Could Potentially Interfere with Vaccine-Induced Immunity 16](#__RefHeading___Toc147556312)

[4.4 Contraindications to Vaccination 16](#__RefHeading___Toc147556313)

[4.5 Indications for Deferral of Vaccination 17](#__RefHeading___Toc147556314)

[4.6 Subject Withdrawal Criteria 17](#__RefHeading___Toc147556315)

[5.0 Vaccine Preparation 18](#__RefHeading___Toc147556316)

[5.1 Supplies 18](#__RefHeading___Toc147556317)

[5.2 Vaccine Storage 18](#__RefHeading___Toc147556318)

[5.3 Vaccine Accountability 18](#__RefHeading___Toc147556319)

[5.4 Disposition of Used/Unused Supplies 18](#__RefHeading___Toc147556320)

[6.0 Study Procedures 18](#__RefHeading___Toc147556321)

[6.1 Screening (Up to 60 Days Prior to Vaccination) 19](#__RefHeading___Toc147556322)

[6.2 Enrollment 19](#__RefHeading___Toc147556323)

[6.3 Randomization 19](#__RefHeading___Toc147556324)

[6.4 Immunization Procedure 19](#__RefHeading___Toc147556325)

[6.5 Clinical Monitoring and Evaluation 20](#__RefHeading___Toc147556326)

[6.6 Volunteer Symptom Diary 23](#__RefHeading___Toc147556327)

[6.7 Laboratory Testing 23](#__RefHeading___Toc147556328)

[6.8 Immunologic Testing 24](#__RefHeading___Toc147556329)

[6.8.1 Antibody Assay (ELISA) 24](#__RefHeading___Toc147556330)

[6.8.2 Transmission Blocking Assay (Membrane Feeds) 24](#__RefHeading___Toc147556331)

[6.9 Use, Storage, and Tracking of Specimens and Data 25](#__RefHeading___Toc147556332)

[7.0 Adverse Events Monitoring and Reporting 25](#__RefHeading___Toc147556333)

[7.1 Definitions 25](#__RefHeading___Toc147556334)

[7.1.1 Adverse Event (AE) 25](#__RefHeading___Toc147556335)

[7.1.2 Serious Adverse Event (SAE) 25](#__RefHeading___Toc147556336)

[7.2 Assessment of Adverse Events 26](#__RefHeading___Toc147556337)

[7.2.1 Identification of AEs 26](#__RefHeading___Toc147556338)

[7.2.2 Determination of Severity 26](#__RefHeading___Toc147556339)

[7.2.3 Association with Receipt of the Study Vaccine 28](#__RefHeading___Toc147556340)

[7.3 Adverse Event Reporting 28](#__RefHeading___Toc147556341)

[7.4 Adverse Event Monitoring 29](#__RefHeading___Toc147556342)

[7.4.1 Medical Monitor 29](#__RefHeading___Toc147556343)

[7.4.2 Safety Monitoring Committee 29](#__RefHeading___Toc147556344)

[7.5 Stopping Criteria 29](#__RefHeading___Toc147556345)

[8.0 Data Collection and Monitoring 30](#__RefHeading___Toc147556346)

[8.1 Source Documentation 30](#__RefHeading___Toc147556347)

[8.2 Study Documentation 30](#__RefHeading___Toc147556348)

[8.3 Retention of Records 30](#__RefHeading___Toc147556349)

[8.4 Protocol Revisions 31](#__RefHeading___Toc147556350)

[8.5 Clinical Investigator’s Brochure 31](#__RefHeading___Toc147556351)

[8.6 Study Monitoring 31](#__RefHeading___Toc147556352)

[9.0 Statistical Considerations 31](#__RefHeading___Toc147556353)

[9.1 General Design 31](#__RefHeading___Toc147556354)

[9.1.1 Description of the Statistical Methods to Be Employed 31](#__RefHeading___Toc147556355)

[9.1.2 Safety 33](#__RefHeading___Toc147556356)

[9.1.3 Immunogenicity Analysis 33](#__RefHeading___Toc147556357)

[9.2 Sample Size 34](#__RefHeading___Toc147556358)

[10.0 Protection of Human Subjects 34](#__RefHeading___Toc147556359)

[10.1 Institutional Review Board/Ethics Committee 34](#__RefHeading___Toc147556360)

[10.2 Informed Consent 34](#__RefHeading___Toc147556361)

[10.3 Risks 34](#__RefHeading___Toc147556362)

[10.3.1 Venipuncture 34](#__RefHeading___Toc147556363)

[10.3.2 Immunization 34](#__RefHeading___Toc147556364)

[10.4 Benefits 35](#__RefHeading___Toc147556365)

[10.5 Confidentiality 35](#__RefHeading___Toc147556366)

[10.6 Compensation 35](#__RefHeading___Toc147556367)

[11.0 References 35](#__RefHeading___Toc147556368)

[Appendix A – Volunteer Recruitment Advertisements 37](#__RefHeading___Toc147556369)

[Appendix B – Volunteer Phone Screen 39](#__RefHeading___Toc147556370)

[Appendix C – Informed Consent Comprehension Exam 42](#__RefHeading___Toc147556371)

[Appendix D – Schedule of Visits 43](#__RefHeading___Toc147556372)

[Appendix E – Volunteer Diary Cards 44](#__RefHeading___Toc147556373)

[Appendix F – Injection Site Reaction Measuring Tool 49](#__RefHeading___Toc147556374)

[Appendix G – Toxicity Table for Grading Laboratory Adverse Events 50](#__RefHeading___Toc147556375)

[Appendix H – Common Toxicity Criteria for Grading Unexpected Adverse Events 51](#__RefHeading___Toc147556376)

Protocol Summary

**Protocol Title:** Phase 1 Study of the Safety and Immunogenicity of PpPfs25/ISA51 and ScPvs25/ISA51: Transmission Blocking Vaccines for *Plasmodium falciparum* and *Plasmodium vivax* Malaria

**Version:** Version 4.0

**Revision History: August 20, 2007;** October 6, 2007**;** January 13, 2006**;** December 19, 2005; November 7, 2005; June 13, 2005; April 26, 2005; February 1, 2005; January 24, 2005; December 8, 2004

**Volunteers:** Healthy malaria-unexposed male and non-pregnant female volunteers 18 to 50 years of age

**Number of Volunteers:** 72 (6 cohorts with 2 placebo/cohort)

**Trial Design:** Single blinded dose escalating clinical trial

**Immunization Schedule:**

| **Cohort** | **Number of Volunteers** | **Immunization Schedule** | |
| --- | --- | --- | --- |
|  |  | **Month 0** | **Month 4** |
| 1 | 12 | A (10) + G (2) | A (10) + G (2) |
| 2 | 12 | B (10) + G (2) | B (10) + G (2) |
| 3 | 12 | C (10) + G (2) | C (10) + G (2) |
| 4 | 12 | D (10) + G (2) | D (10) + G (2) |
| 5 | 12 | E (10) + G (2) | E (10) + G (2) |
| 6 | 12 | F (10) + G (2) | F (10) + G (2) |
| Total | 72 | A: 5 μg PpPfs25/ISA51  B: 20 μg PpPfs25/ISA51  C: 80 μg PpPfs25/ISA51  D: 5 μg ScPvs25/ISA51  E: 20 μg ScPvs25/ISA51  F: 80 μg ScPvs25/ISA51  G: PBS/ISA51 – placebo | |

.

**Product Description:** The vaccine formulations to be studied were produced as recombinant proteins expressed in *Pichia pastoris* and *Saccharomyces cerevisiae,* for PpPfs25 and ScPvs25, respectively. Bulk antigens were purified from the culture supernatant by a combination of metal affinity, hydrophobic interaction and size-exclusion chromatography. The purified drug substances were individually emulsified with Montanide® ISA51 (SEPPIC, France).

**Time Period:** A total of 90 weeks including vaccinations, study procedures, and follow-up of all volunteers. Each volunteer will be followed for a total of 78 weeks (maximum of 82 weeks including screening).

# Introduction

## Background

As reported by the World Health Organization (WHO) in 2002, the worldwide incidence of malaria is approximately 300 million clinical cases annually, with approximately one million deaths per year attributed to malaria alone or in combination with other diseases [1]. The development of a safe and effective vaccine that prevents the transmission of *Plasmodium falciparum* and *Plasmodium vivax* would be an important addition to the current methods for controlling the spread of malaria parasites. Most of the malaria mortality occurs in sub‑Saharan Africa and in children under 5 years of age. Of the four species of malaria parasite that infect humans, *P. falciparum* is responsible for the majority of these deaths. However, outside of Africa, most of the malaria is caused *by P. vivax*; although these cases result in few deaths, they represent a major cause of morbidity and lead to a significant impact on the quality of life for more than 1.5 billion people [2]. Mounting drug resistance of the malaria parasite, as well as widespread resistance of mosquitoes to insecticides, make these control strategies increasingly unrealistic. A vaccine that would reduce both mortality and morbidity secondary to *P. falciparum* and *P. vivax* infection would be a valuable resource in the fight against this disease.

## Transmission Blocking Vaccines

Of the different stages of the complex malaria life cycle, the mosquito stage is a potential target of immune intervention, albeit indirectly. Since only the mosquito stages of the parasite carry the infection from humans to mosquitoes, and since man is the only known reservoir of the disease, disruption of the development of the mosquito-stage parasites in the mosquito midgut should diminish, and perhaps even completely prevent the spread of malaria parasites. A vaccine designed to prevent oocyst development in the mosquito by eliciting transmission‑blocking antibodies in the vertebrate host would protect other individuals in the vicinity from becoming infected from the immunized person.

Thus, despite no immediate role in personal protection, transmission-blocking vaccines are a potentially powerful component of a multifaceted public health approach to controlling or eliminating malaria. An effective malaria transmission-blocking vaccine could have an impact analogous to the role that vaccines such as smallpox, polio, and measles have had in the eradication of their respective diseases by preventing the transmission of pathogens from individuals to the community. The efficacy and coverage of a malaria transmission-blocking vaccine required to have an impact will depend on the local transmission rate. Intensive intervention with a transmission-blocking vaccine, in combination with other measures such as impregnated bed nets, chemotherapy, and/or vector control programs, may eliminate malaria in such a community.

## Vaccine Description

### Drug Substances

Both recombinant PpPfs25 and ScPvs25 are highly purified proteins. The recombinant Pfs25 protein was expressed in *Pichia pastoris* and the recombinant Pvs25 was expressed in *Saccharomyces cerevesiae* and purified from culture supernatants by a combination of metal affinity, hydrophobic interaction and size-exclusion chromatography. The purification process was designed to separate full-length, correctly folded product from degraded material as well as nonproduct related impurities. The bulk Drug Substances were supplied in sterile phosphate buffered saline (pH 7.4) containing no preservatives. Both Drug Substances were manufactured under cGMP conditions at the Walter Reed Army Institute of Research (WRAIR), Bioproduction Facility (Silver Spring, Maryland). Prior to release, the drug substances underwent comprehensive quality control analysis to ensure specifications were met.

### Montanide® ISA51

Montanide® ISA51 (SEPPIC, France) is a water-in-oil adjuvant. Montanide® ISA51 has been used as an experimental adjuvant in humans in the United States and elsewhere. While Montanide® ISA51 is not a component in any licensed vaccines in the U.S., experience with human trials indicates that it is an effective immune enhancer and at the protein doses to be used in this trial, has generally been well tolerated.

### Vaccine Formulation

Two experimental malaria vaccines will be tested in this vaccine trial at 3 doses: 5, 20, and 80 μg of PpPfs25/ISA51 and 5, 20, and 80 μg of ScPvs25/ISA51. The clinical trial site pharmacist will provide single dose vials of vaccine or placebo (phosphate buffered saline (PBS)/ISA51) to the clinical trial staff on the day of scheduled vaccinations. Each vial will contain 1.0 mL of vaccine (or placebo) in a stoppered, non-siliconized glass vial which has been designed for delivery of a 0.5 mL dose. The 80 μg doses of both PpPfs25/ISA51 and ScPvs25/ISA51, as well as the placebo, were produced by the Pharmaceutical Development Section, Clinical Center, NIH and will be supplied to the clinical trial site as ready for use. The clinical trial site pharmacy will be responsible for preparation of the 5 and 20 μg doses of vaccine by diluting the supplied 80 μg vaccines with placebo (PBS/ISA51) as per an established procedure. Clinical staff will draw 0.5 mL of test article (vaccine or placebo) from these unit dose vials into a 1.0 mL syringe using a needle of appropriate gauge and length on the day of vaccination.

## Rationale

### Preclinical Experience

PpPfs25 and ScPvs25 alone, in the absence of any adjuvant, were not sufficiently immunogenic when administered to mice and rabbits. This demonstrated the need for an adjuvant in order to achieve a significant immune response while administering a minimal amount of antigen. Among all adjuvants tested, Montanide®ISA51 provided the strongest and most consistent adjuvanticity to antigens in both mice and rabbits. In addition, rabbits immunized with the Montanide® ISA51 formulations developed a sustained high level antibody response, which we feel is important for a successful transmission blocking vaccine. This finding is consistent with the “depot” effect that has been observed in water-in-oil emulsions.

Antibodies induced by the Montanide®ISA51 formulation were capable of blocking parasite development in mosquitoes in an *ex vivo* membrane feeding assay.

To support the clinical use of the Montanide®ISA51 formulation, a 14-week GLP toxicology study was conducted in rabbits to assess the development of adverse events which might occur as a result of test article administration. Rabbits received 4 immunizations with the highest dose of ScPvs25/ISA51 or PpPfs25/ISA51 intended for humans. No vaccine-related changes in mortality, clinical observations, cage side observation, Draize observations, body weights and weight changes, food consumption, ophthalmology, organ weights and weight ratios, gross pathology, clinical pathology, or gross necropsy observations were observed. Histopathology revealed inflammation at the injection sites. While the characteristics varied slightly between groups, this inflammation was felt to be the result of the Montanide®ISA51 adjuvant, and the addition of the antigens, PpPfs25 or ScPvs25, did not considerably alter this profile. The toxicology study report concluded that, “under these conditions, repeated intramuscular injection of 80 μg of the PpPfs25 or ScPvs25 antigens formulated with Montanide®ISA51 adjuvant did not result in obvious systemic or local toxicity in New Zealand White Rabbits”.

The preclinical studies, including the GLP toxicology study, demonstrate that PpPfs25/ISA51 and ScPvs25/ISA51 were immunogenic and well tolerated. Antibodies induced by the drug products were functionally active in blocking parasite development in mosquitoes in an *ex vivo* assay. These results support proceeding with a Phase 1 trial in humans with PpPfs25/ISA51 and ScPvs25/ISA51.

### Clinical Experience with PpPfs25H/ISA51 and ScPvs25H/ISA51

The vaccine formulations in this protocol have not been tested in human trials. The ScPvs25 recombinant protein has been tested when formulated with Alhydrogel® in a Phase 1 human trial and the vaccine was well tolerated. Ten volunteers in each of three dose groups (5 µg, 20 µg, or 80 µg) were vaccinated by intramuscular injection in an open label study at 0, 28 and 180 days. No vaccine-related serious adverse events were observed. The majority of adverse events causally related to vaccination were mild or moderate in severity. Injection site tenderness was the most commonly observed adverse event.

A related recombinant antigen TBV25H, derived from Pfs25, has been used in a Phase 1 human trial with 250 μg administered on Days 0, 28, and 56. The dose used in the TBV25H trial is three times the maximum dose of 80 μg that will be used in this trial. A hypersensitivity reaction was observed in one of seven subjects who received the vaccine following the third immunization with 250 μg of TBV25H. This reaction involved swelling at the injection site 30 minutes following the third injection, followed by swelling in the contralateral arm (the site of the second injection) 1 hour after receipt of the third injection. The TBV25H formulation contained approximately 40% free antigen, and available data from studies in mice indicate that the hypersensitivity reaction with this formulation was due to the free antigen. The reaction seen with TBV25H is not anticipated with the formulations to be tested in this protocol as the formulation to be tested in this trial is an emulsion. The antigen is therefore localized to small water droplets contained in the oil portion of the emulsion.

### Clinical Experience with Montanide® ISA51 and Related Adjuvants

Montanide® ISA51 is chemically similar to incomplete Freund’s adjuvant (IFA). There is extensive experience with the use of IFA in humans. Since 1945, more than one million people have been vaccinated with IFA in various forms. IFA has been associated with a high incidence of severe local reactions in certain experimental vaccines.

More than 4,500 individuals have received more than 45,000 doses of vaccines containing Montanide®ISA51. The immunogens used in these studies included peptides (26 trials), proteins (12 trials) and ganglioside-protein complexes (3 trials). These vaccines were generally well tolerated. Most reported adverse reactions were transient local reactions (55% of 1862 subjects in 27 trials with available data) that resolved within 48-72 hours. The most common adverse reactions were local swelling and pain with or without fever. Mild to moderate (Grade 1or 2) injection site nodules, sometimes termed granulomas were reported in 8 of 41 trials, affecting 83 of 187 subjects. None required surgical resection, and results of diagnostic biopsies were not provided. All resolved within weeks without medical intervention.

Abscesses at injection sites have been reported in three published trials. In one study, “cold abscesses” at the injection sites were reported in 2 of 122 recipients of immunogen plus Montanide®ISA51 in a large trial of rIFN-α protein [3]. These abscesses occurred after the first injection and did not require surgical drainage; however, the volunteers were given no further immunizations. In a second trial, one patient of 21 who received multiple injections of 200 mg of NeuGcGM3 ganglioside in ISA51 developed a “small abscess” at the injection site. The treatment for this was not reported [4]. In a third trial, with inactivated HIV as an immunogen (containing 100 μg of P24 antigen) carried out in Thailand, 1 of 30 HIV infected vaccinees reported a cyst at the site of injection (1/120 injections) at week 12. The relationship to the fourth vaccination, also scheduled for week 12 was not clear and the details of the workup were not provided. The study clinician diagnosed the lesion as a sterile cyst, and it resolved without any treatment by week 16 of the study [5].

There are 71 ongoing or completed clinical trials (as of 30 November, 2004) in which Montanide®ISA51 is used as an adjuvant (reference [http://clinicaltrials.gov/](http://clinicaltrials.gov/ct/gui) ). These include data from trials which have been published, others that are no longer recruiting or are completed (37 trials), and ongoing studies (34 trials), which are projected to recruit a cumulative 10,060 subjects when completed. Local reactions at the injection site are the most common findings, estimated by one principal investigator to be present in virtually all recipients, with approximately 15% of Montanide®ISA51 recipients having moderate (Grade 2) local reactions. The reactions were described as induration and occasional superficial ulcerations at the injection site. The vaccine was delivered subcutaneously in these trials. These reactions occurred in both initial and subsequent injections (in different individuals), and resolved within days without surgery or antibiotic treatment.

Abscess formation has been observed in one unpublished study, in which Montanide®ISA51 was used as an adjuvant with an HIV-derived, HLA binding peptide, at a dose range of 1-4 mg of the immunogen in 2 mL of Montanide®ISA51 formulation and delivered and in 4 x 0.5 mL injections) to healthy volunteers (B. Graham, personal communication). In this trial, cold abscesses occurred in four of twenty recipients of Montanide®ISA51 two to four weeks after vaccination. These reactions were classified as Grade 3 local reactions. Volunteers with the abscesses had received either 1 mg or 4 mg of the immunogen. Two abscesses occurred after the first injection and two occurred after the second injection. All four required surgical drainage, but showed no evidence of infection.

## Clinical Development Plan

The first Phase 1 trial will be carried out in the United States. The study will be a single blinded, dose-escalating Phase 1 clinical trial in healthy adult volunteers designed to evaluate the safety, reactogenicity and immunogenicity of the PpPfs25/ISA51 and ScPvs25/ISA51 in malaria-naive individuals. The maximum human dose of 80 µg/0.5 mL administered intramuscularly was tested in a rabbit toxicology study, and no clinically significant adverse effects were observed. Therefore, this dose was deemed to be reasonable.

If either vaccine candidate is determined to be immunogenic then the antibodies produced in this trial may form the basis of an international reference standard for transmission blocking vaccine development. Assuming the individual components (PpPfs25 and ScPvs25) demonstrate safety and immunogenicity, the next step would be to combine the antigens into a single vaccine. The combination would be optimized in a series of U.S. Phase 1 trials for safety and immunogenicity prior to taking the vaccine to the field. A Phase 1 trial of the combination vaccine formulation will be repeated in healthy, malaria-exposed adults in a malaria-endemic region, given the possibility that the safety of this vaccine formulation may be different in such a population. Provided no safety concerns become apparent, age de-escalation, Phase 2, and eventually Phase 3 clinical trials will be undertaken in malaria-endemic areas.

### Participation of Children

The vaccine candidates being tested in this protocol have not yet been tested in humans. There are insufficient data to judge the potential risk in children. Once safety is established in adults in the United States and then in an endemic region, we intend to age de-escalate to children in malaria endemic regions.

# Objectives

## Primary Objective

To assess the safety, reactogenicity, and immunogenicity of the PpPfs25/ISA51 and ScPvs25/ISA51 malaria vaccines.

## Secondary Objectives

1. To assess and compare the duration of specific antibody response over an 18 month period.
2. To measure the effect of boosting at 4 months on specific antibody levels.

## Tertiary Objectives

1. To measure the ability of vaccine induced antibody to inhibit oocyst development in a mosquito membrane feeding assay
2. To determine the relationship between antibody levels and degree of transmission blocking in a membrane feeding assay
3. This study will form the basis of further transmission blocking vaccine development, including the establishment of human standards for immune assays.

# Study Design

## Overall Design

This trial will be performed under the ICH and current Good Clinical Practices guidelines and in compliance with 21 CFR 50 & 56. The study is a single blinded (blinded to volunteers) Phase 1 dose-escalating clinical trial in healthy adult volunteers designed to evaluate the safety, reactogenicity and immunogenicity of PpPfs25 and ScPvs25 emulsified with Montanide® ISA51. Volunteers will be recruited and screened; those determined to be eligible, based on the inclusion and exclusion criteria described in **Section 4.0** in this protocol, will be enrolled in the study. After providing written informed consent, volunteers will undergo eligibility screening, including medical history, physical examination, hematology testing, liver and renal function testing, HIV, Hepatitis B and C screening and urinalysis. Urine pregnancy testing will be performed on female volunteers; and females will be counseled to avoid becoming pregnant during the study. Clinically significant abnormalities will be reviewed with volunteers and referral for follow-up care will be provided. For eligible volunteers, the Day 0 visit will be scheduled for receipt of the first dose of vaccine. Vaccinated volunteers will be observed for immediate reactions following each vaccination for 30 minutes. Volunteers will return to the clinic on Days 1, 3, 7, 14 and 21 following each vaccination for clinical assessment. See **Tables 1 and 2** for a tabular description of the vaccination schedule.

Seventy two (60 vaccine and 12 placebo) volunteers will be enrolled and assigned to one of six cohorts as outlined in **Tables 1 & 2**. All vaccines will contain the same amount of Montanide® ISA51. As with other recombinant protein vaccines, hypersensitivity reactions would be expected to occur within the first 24 hours after receipt of the vaccine, and other severe local or systemic reactions within 72 hours, and possibly up to 3 weeks post-vaccination. The vaccinations will be staggered within one dose cohort such that four volunteers will be vaccinated three weeks prior to the remaining 8 volunteers. Therefore, 35 days of safety data will be available for the first 4 vaccinees and 21 days of safety data for the remaining 8 volunteers post-vaccination from the lower dose cohort for review by the Safety Monitoring Committee (SMC) prior to dose escalation. Dose escalation will not occur less than 3 weeks from the remaining 8 volunteers in the lower dose cohort being vaccinated. The trial will not proceed to the next dose cohort if, in the clinical judgment of the SMC, the next higher dose would pose an unacceptable safety risk to the volunteers. The PpPfs25 and ScPvs25 formulations will be considered individually by the SMC prior to dose escalation. The following table represents the earliest projected vaccination dates based on the safety intervals described above. These represent only tentative dates and may change based on recommendations of the Safety Monitoring Committee and/or the study staff.

Tables 1 and 2: Vaccination Schedule

| **PpPfs25/ISA51 Vaccine Dose Escalation Schedule** | | | | |  | **ScPvs25/ISA51 Vaccine Dose Escalation Schedule** | | | | |
| --- | --- | --- | --- | --- | --- | --- | --- | --- | --- | --- |
|  |  | Cohort A^1^: (n=12) | Cohort B^1^: (n=12) | Cohort C^1^: (n=12) |  |  |  | Cohort A^2^: (n=12) | Cohort B^2^: (n=12) | Cohort C^2^: (n=12) |
| Date | Week | 5µg | 20µg | 80µg |  | Date | Week | 5µg | 20µg | 80µg |
| 5/23/05 | 0 | **Vax 1 (n=4)** |  |  |  | 6/13/05 |  |  |  |  |
| 5/30/05 | 1 |  |  |  |  | 7/11/05 | 0 | **Vax 1 (n=4** |  |  |
| 6/6/05 | 2 | **Vax 1 (n=5)** |  |  |  | 7/18/05 | 1 |  |  |  |
| 6/13/05 | 3 |  |  |  |  | 7/25/05 | 2 |  |  |  |
| 6/20/05 | 4 | **Vax 1 (n=3)** |  |  |  | 8/1/05 | 3 | **Vax 1 (n=4)** |  |  |
| 8/22/05 |  |  |  |  |  | 8/22/05 |  | **Vax 1 (n=4)** |  |  |
| 9/26/05 | 18 | **Vax 2 (n=4)** |  |  |  | 9/26/05 | 11 |  | **Vax 1 (n=4** |  |
| 10/3/05 | 19 |  |  |  |  | 10/3/05 | 12 |  |  |  |
| 10/10/05 | 20 |  |  |  |  | 10/10/05 | 13 |  |  |  |
| 10/17/05 | 21 | **Vax 2 (n=5)** |  |  |  | 10/17/05 | 14 |  | **Vax 1 (n=8)** |  |
| 10/24/05 | 22 |  |  |  |  | 10/24/05 | 15 |  |  |  |
| 10/31/05 | 23 |  |  |  |  | 10/31/05 | 16 |  |  |  |
| 11/7/05 | 24 | **Vax 2 (n=3**) |  |  |  | 11/7/05 | 17 | **Vax 2 (n=4)** |  |  |
| 11/14/05 | 25 |  | **Vax 1 (n=4)** |  |  | 11/14/05 | 18 |  |  | **Vax 1 (n=4)** |
| 11/21/05 | 26 |  |  |  |  | 11/21/05 | 19 |  |  |  |
| 11/28/05 | 27 |  |  |  |  | 11/28/05 | 20 | **Vax 2 (n=4)** |  |  |
| 12/5/05 | 28 |  | **Vax 1 (n=8)** |  |  | 12/5/05 | 21 |  |  | **Vax 1 (n=8)** |
| 12/12/05 | 29 |  |  |  |  | 12/12/05 | 22 |  |  |  |
| 12/19/05 | 23 |  |  |  |  | 12/19/05 | 23 | **Vax 2 (n=4)** |  |  |
| 1/23/06 | 28 |  |  |  |  | 1/23/06 | 28 |  | **Vax 2 (n=4)** |  |
| 2/14/06 | 31 |  |  |  |  | 2/14/06 | 31 |  | **Vax 2 (n=8)** |  |
| 3/6/06 | 34 |  |  |  |  | 3/6/06 | 34 |  |  |  |
| 3/13/06 | 35 |  | **Vax 2 (n=4)** |  |  | 3/13/06 | 35 |  |  | **Vax 2 (n=4)** |
| 3/20/06 | 36 |  |  |  |  | 3/20/06 | 36 |  |  |  |
| 3/27/06 | 37 |  |  |  |  | 3/27/06 | 37 |  |  |  |
| 4/3/06 | 38 |  | **Vax 2 (n=8)** |  |  | 4/3/06 | 38 |  |  | **Vax 2 (n=8)** |
| 4/17/06 | 40 |  |  |  |  | 4/17/06 | 40 |  |  |  |
| 4/24/06 | 41 |  |  | **Vax 1 (n=4)** |  | 4/24/06 | 41 |  |  |  |
| 5/15/06 | 44 |  |  | **Vax 1 (n=8)** |  |  |  |  |  |  |
|  |  |  |  |  |  |  |  |  |  |  |
| 8/21/06 | 58 |  |  | **Vax 2 (n=4)** |  |  |  |  |  |  |
| 9/18/06 | 61 |  |  | **Vax 2 (n=8)** |  |  |  |  |  |  |

1. PpPfs25/ISA51 (n=10) or PBS/ISA51 (n=2)

2. ScPvs25/ISA51 (n=10) or PBS/ISA51 (n=2)

* Within each cohort, the volunteers will be staggered such that 4 volunteers (3 volunteers will receive PpPfs25/ISA51 or ScPvs25/ISA51 and 1 will receive placebo) will be vaccinated 3 weeks prior to the remaining 8 volunteers (7 volunteers will receive PpPfs25/ISA51 or ScPvs25/ISA51 and 1 will receive placebo). The lightly shaded area represents the portion of the study that has been completed.

## Sample Size and Estimated Duration of Study

Seventy-two volunteers will be enrolled. This trial is expected to last for a total of 90 weeks. Each volunteer will be followed for 78 weeks from the time of the first injection, for a maximum of 82 weeks including screening.

## Cohort Allocation

Volunteers will be randomized to one of six vaccine cohorts. Within each vaccine cohort, 2 volunteers will be randomized to receive placebo, and 10 volunteers to receive vaccine.

# Selection and Enrollment of Volunteers

## Inclusion Criteria

1. Males or females between 18 and 50 years, inclusive.
2. Good general health as a result of review of medical history and/or clinical tests at screening.
3. Available for the duration of the trial (78 weeks).
4. Willingness to participate in the study as evidenced by signing the informed consent document.

## Exclusion Criteria

1. Pregnancy as determined by a positive urine human chorionic gonadotrophin (ß-hCG), if female.
2. Volunteer unwilling to use reliable contraception methods for the duration of the trial, if female.

(Reliable methods of birth control include: pharmacologic contraceptives including oral, parenteral, and transcutaneous delivery; condoms with spermicide, diaphragm with spermicide, surgical sterilization, intrauterine device, abstinence, and post-menopause)

1. Currently breast-feeding (if female).
2. Behavioral, cognitive, or psychiatric disease that in the opinion of the investigator affects the ability of the volunteer to understand and cooperate with the study protocol.
3. Laboratory evidence of liver disease (aspartate aminotransferase [AST] and/or alanine aminotransferase [ALT] greater than the upper limit of normal of the testing laboratory).
4. Laboratory evidence of renal disease (serum creatinine greater than the upper limit of normal of the testing laboratory).
5. Laboratory evidence of hematologic disease (absolute neutrophil count < 1,500/mm^3^; hemoglobin less than the lower limit of normal of the testing laboratory, by sex; or platelet count < 140,000/mm^3^).
6. Evidence of clinically significant neurologic, cardiac, pulmonary, hepatic, endocrine, rheumatologic, autoimmune, or renal disease by history, physical examination, and/or laboratory studies including urinalysis (greater than trace protein, or any glucose on urine dip will be confirmed negative prior to enrollment).
7. Other condition that in the opinion of the investigator would jeopardize the safety or rights of a volunteer participating in the trial or would render the subject unable to comply with the protocol.
8. Participation in another investigational vaccine or drug trial within 30 days of starting this study, or while this study is ongoing.
9. Volunteer has had medical, occupational, or family problems as a result of alcohol or illicit drug use during the past 12 months
10. History of a severe allergic reaction or anaphylaxis.
11. Positive ELISA and confirmatory Western blot tests for HIV-1.
12. Positive ELISA and confirmatory immunoblot tests for HCV.
13. Positive HBsAg by ELISA.
14. Known immunodeficiency syndrome.
15. Use of corticosteroids (excluding topical or nasal) or immunosuppressive drugs within 30 days of starting this study or while the study is ongoing.
16. Receipt of a live vaccine within past 4 weeks or a killed vaccine within past 2 weeks prior to entry into the study.
17. History of a surgical splenectomy.
18. Receipt of blood products within the past 6 months.
19. Previous receipt of an investigational malaria vaccine.
20. History of a known allergy to nickel.

Note: For this study, prior history of malaria or a recent visit to a malaria endemic area are not exclusion criteria.

## Treatments That Could Potentially Interfere with Vaccine-Induced Immunity

The following criteria should be checked prior to each vaccination. If any become applicable during the study, the volunteer will be excluded from receiving further doses of the study vaccine and will not be included in the immunogenicity evaluations after the time of exclusion. The volunteer will, however, be encouraged to remain in the study for further safety evaluation for doses already received.

1. Use of any investigational drug or investigational vaccine other than the study vaccine during the study period.
2. Administration of chronic (defined as more than 14 days) immunosuppressants or other immune-modifying drugs 6 months prior to vaccination. (Topical and nasal steroids are allowed.)
3. Administration of a licensed vaccine 14 days before or after each vaccination.
4. Administration of immunoglobulins and/or any blood products up to 30 days after the last dose of vaccine.

## Contraindications to Vaccination

The following criteria should be checked prior to each immunization and are contraindications to further immunization. However, the volunteer will be encouraged to remain in the study for safety evaluation for doses already received. Should a female volunteer become pregnant during the course of the study, she will be referred to an appropriate specialist and followed for the duration of the pregnancy.

1. Hypersensitivity reaction following administration of the study vaccine.
2. Pregnancy, as determined by a positive urine ß-hCG.

## Indications for Deferral of Vaccination

The following AEs constitute grounds for deferral of vaccine administration at that point in time; if any one of these AEs occurs at the time scheduled for vaccination, the volunteer may be vaccinated at a later date, within the allowable time interval specified in the protocol, or withdrawn at the discretion of the investigator. The volunteer must be followed until resolution of the event as with any AE. If the volunteer is withdrawn from the study, he/she will be encouraged to continue in the safety evaluation for the duration of the study.

1. Oral temperature > 37.5°C at the time of vaccination will warrant deferral of immunization until fever and symptoms resolve.
2. Any other condition that in the opinion of the investigator poses a threat to the individual if immunized or that may complicate interpretation of the safety of the vaccine following immunization.

Such individual(s) will be followed in the clinic until the symptoms resolve or the window for immunization expires. No further vaccination will be performed if the volunteer does not recover (oral temperature ≤ 37.5°C and/or lack of symptoms) within the vaccination window described in Section 6.5 of this protocol. The volunteer, however, will be followed for safety and immunogenicity. If the individual meets any of the above criteria for deferral on the day of first immunization, the investigator may elect to exclude the volunteer from further participation in the study. Eligible alternates will then be vaccinated instead.

## Subject Withdrawal Criteria

A volunteer will not be considered to have completed the trial if any of the following reasons apply. However, any volunteer who has received at least one dose of vaccine will be encouraged to remain in the study for safety evaluation for the duration of the study.

1. *Research terminated by sponsor or investigator* – applies to the situation where the entire study is terminated by the sponsor, or investigator for any reason.
2. *Withdrawal of consent* – applies to a subject who withdraws consent to participate in the study for any reason.
3. *Noncompliant with protocol* – applies to a volunteer who does not comply with protocol-specific visits or evaluations, on a consistent basis, such that adequate follow-up is not possible and the volunteer’s safety would be compromised by continuing in the trial. Additionally, this applies to a volunteer who is lost to follow-up and is not reachable by telephone or other means of communication, and therefore not able to be located. In the event that a volunteer becomes incarcerated during the course of this study, every attempt will be made to continue to monitor for safety for the duration of the study.

4*. Other* – is used when previous categories do not apply and a written explanation is required.

# Vaccine Preparation

## Supplies

Research products for this protocol will be supplied to the study site pharmacist by the Pharmaceutical Development Section (PDS), Pharmacy Department, Clinical Center, National Institutes of Health (NIH), where the vaccines were formulated and vialed. Vaccines will be transported at 2^o^C to 8^o^C from the PDS at the NIH to the study site pharmacy with a temperature recording device. Upon receipt of vaccine supplies, the study site pharmacist will ensure that the temperature was maintained between 2^o^C to 8^o^C using the temperature recording device and it’s associated computer program. The study site pharmacy will be responsible for maintaining the appropriate supply of vaccine and will request additional vaccine in writing through the IND sponsor.

PpPfs25/ISA51 and ScPvs25/ISA51 malaria vaccines are supplied as a white emulsion in single‑dose vials containing 160 µg/mL. Additionally, the placebo (PBS/ISA51) will be supplied as a white emulsion in single dose vials. Each 2.0 mL vial contains 1.0 mL emulsion, of which 0.5 mL is the intended volume to be injected. 0.5 mL of vaccine contains 0.25 mL phosphate-buffered saline and 0.25 mL Montanide® ISA51. The placebo contains 0.25 mL of PBS and 0.25 mL Montanide® ISA51. The Drug Product conforms to established requirements for sterility, safety and identity. The 5 µg and 20 µg vaccines will be prepared in the trial site pharmacy by diluting the high dose (160 µg/mL) vaccine with placebo on the day of vaccination.

## Vaccine Storage

The PpPfs25/ISA51 and ScPvs25/ISA51 malaria vaccines should be maintained at 2^o^C to 8^o^C until just prior to administration. Vaccine should NOT be frozen at any time. Single-dose vials should be stored in the upright position. Vaccine will be prepared by the trial site pharmacist, and must be used within 4 hours and kept refrigerated at 2^o^C to 8^o^C until just before use.

## Vaccine Accountability

The trial site pharmacists are responsible for maintaining an accurate inventory and accountability record of vaccine supplies for this study. Partially used vials may not be administered to other volunteers.

## Disposition of Used/Unused Supplies

After administration of a vaccine dose, the single-dose vial will be returned to the trial site pharmacy, and vials will be accounted for and stored until monitoring by the IND Sponsor. Used vials may be disposed of according to study site protocol after monitoring has been completed. Final disposition of unused vaccine supplies will be determined by the Malaria Vaccine Development Branch, NIH, in conjunction with the IND Sponsor.

# Study Procedures

The following sections provide a detailed listing of the procedures and studies to be performed in this protocol at designated time points. The total volume of blood (approximately 365 mL) to be drawn over the duration of the trial is less than volume collected when donating a single unit of blood and should not compromise the health of trial volunteers.

## Screening (Up to 60 Days Prior to Vaccination)

1. Explain the study and Informed Consent to the volunteer.
2. Ensure the subject has signed the Informed Consent and received a signed copy of the Informed Consent.
3. Explain the HIV Informed Consent and ensure the subject has signed the HIV Informed Consent.
4. Elicit a complete medical history, including menstrual and contraceptive history and/or history of surgical sterility for female subjects.
5. Administer a complete physical examination.
6. Obtain approximately 25 mL of blood for hematology, biochemistry, blood type & screen, serologic tests for viral hepatitis and HIV in all volunteers.
7. Obtain urine for urine dipstick testing, as well as urine β-hCG testing in females.
8. Counsel females to avoid becoming pregnant during the study.

## Enrollment

Volunteers will respond to advertisements distributed by the clinical trial site in local newspapers and posted flyers (See **Appendix A**). A screening visit will be scheduled after an initial contact screen by clinical trial staff, consisting of background information of the trial (**Appendix B**). During this initial screening visit, the volunteer will read the consent form and be encouraged to ask questions, and then take a multiple-choice questionnaire to evaluate consent comprehension (**Appendix C**). The volunteer must answer all questions correctly prior to being eligible for enrollment. Study staff will use incorrect answers from the questionnaire to identify those areas of the informed consent form that need further review with the volunteer. This will help ensure the volunteer has sufficient understanding before the consent form is signed. The volunteer may either sign the consent form during the screening visit or return after further consideration. Seventy two volunteers will be enrolled and assigned to one of 6 vaccine groups (5 µg, 20 µg or 80 µg of PpPfs25/ISA51 or ScPvs25/ISA51). There will be 10 volunteers in each group randomized to receive vaccine and 2 volunteers in each group randomized to receive placebo. Therefore, each of the 6 groups will have 12 volunteers each.

## Randomization

Volunteers will be randomly assigned to receive test article (PpPfs25/ISA51 or ScPvs25/ISA51) vaccine or placebo. Treatment assignment will be done by random number generator. A master log of treatment assignment will be maintained in a record separate from other study records. This log will be kept in the Center for Immunization Research laboratory in the School of Public Health. It will be kept in a locked room with limited access. The volunteers will be informed of their treatment assignment at their day 556 visit.

## Immunization Procedure

Volunteers will receive two immunizations; the second immunization will be given four months after the first. Vaccine will be prepared by the trial site pharmacist, and must be used within 4 hours. Vaccine must be kept refrigerated at 2^o^C to 8^o^C until just before use. 0.5 mL will be delivered by IM injection in the deltoid muscle with a 21‑gauge needle of appropriate length after preparation of the site with alcohol. Successive vaccinations will be given in alternating arms.

## Clinical Monitoring and Evaluation

See **Appendix D** for a tabular representation of study procedures. In addition to the procedures listed below, photographs of the injection may be taken at baseline and on follow-up visits to better assess and evaluate local reactogenicity.

Study Day 0 (Day of First Vaccination)

1. Verify that Informed Consent was obtained.
2. Verify that all applicable eligibility criteria have been met.
3. Perform abbreviated history and physical exam, focusing on any acute complaints.
4. Obtain approximately 35 mL of blood for hematology, biochemistry, antibody ELISA and membrane feed assay.
5. For females, obtain a urine sample for β-hCG testing. Ensure that test is negative before vaccinating; a positive test will exclude the volunteer from the trial.
6. Record vital signs (blood pressure, temperature, heart rate, and respiratory rate).
7. Administer the vaccine.
8. Observe for at least 30 minutes after vaccination to evaluate for immediate adverse reactions.
9. Education by study staff during 30-minute post-immunization wait period describing proper use of digital thermometers, injection-site reaction measurement and malaria vaccine side-effect diaries. Study staff will also discuss signs and symptoms of potential adverse events (AEs).
10. Distribute Days 0-6 diary card.

Study Day 1

1. Perform basic history and physical exam (including injection site), emphasizing examination of any acute complaints.
2. Record vital signs.

Study Day 3

1. Perform basic history and physical exam (including injection site), emphasizing examination of any acute complaints.
2. Record vital signs.
3. Obtain approximately 10 mL of blood for hematology and biochemistry tests.

Study Day 7 +/- 1

1. Perform basic history and physical exam (including injection site), emphasizing examination of any acute complaints.
2. Record vital signs.
3. Collect Days 0-6 diary card.
4. Draw approximately 3.5 mL of blood for CBC and differential
5. Distribute Days 7-13 diary card.

Study Day 14 +/- 2

1. Perform basic history and physical exam (including injection site), emphasizing examination of any acute complaints.
2. Record vital signs.
3. Obtain approximately 15 mL of blood for hematology, biochemistry, and antibody ELISA.
4. Collect Days 7-13 diary card.
5. Distribute Days 14 – 20 diary card.

Study Day 21 +/- 2

1. Perform basic history and physical exam (including injection site), emphasizing examination of any acute complaints.
2. Record vital signs.
3. Collect Days 14 – 20 diary card.

Study Day 30 +/- 7(1 month after first vaccination)

1. Perform basic history and physical exam (including injection site), emphasizing examination of any acute complaints.
2. Record vital signs.
3. Obtain approximately 5 mL of blood for antibody ELISA.

Study Day 60 +/- 7 (2 months after first vaccination)

1. Perform basic history and physical exam (including injection site), emphasizing examination of any acute complaints.
2. Record vital signs.
3. Obtain approximately 35 mL of blood for hematology biochemistry, antibody ELISA & membrane feeding assay.

Study Day 90 +/- 7 (3 months after first vaccination)

1. Perform basic history and physical exam (including injection site), emphasizing examination of any acute complaints.
2. Record vital signs.
3. Obtain approximately 5 mL of blood for antibody ELISA.

Study Day 120 +/- 7 (Day of Second Vaccination)

1. Perform basic history and physical exam (including injection site), emphasizing examination of any acute complaints.
2. Obtain approximately 35 mL of blood for hematology, biochemistry, antibody ELISA, and membrane feeding assay
3. For females, obtain a urine sample for β-hCG testing. Ensure that test is negative before vaccinating; a positive test will exclude the volunteer from the trial.
4. Record vital signs (blood pressure, temperature, heart rate and respiratory rate).
5. Administer the vaccine.
6. Observe for at least 30 minutes after vaccination to evaluate for immediate adverse reactions.
7. Education by study staff during 30-minute post-immunization wait period describing proper use of digital thermometers, injection-site reaction measurement tools and patient diaries. Study staff will also discuss signs and symptoms of potential AEs.
8. Distribute Days 0-6 diary card.

Study Day 121 (1 day after Second Vaccination)

1. Perform basic history and physical exam (including injection site), emphasizing examination of any acute complaints.
2. Record vital signs.

Study Day 123 (3 days after Second Vaccination)

1. Perform basic history and physical exam (including injection site), emphasizing examination of any acute complaints.
2. Record vital signs.
3. Obtain approximately 10 mL of blood for hematology and biochemistry tests.

Study Day 127 +/- 1 (7 days after Second Vaccination)

1. Perform basic history and physical exam (including injection site), emphasizing examination of any acute complaints.
2. Record vital signs.
3. Collect Days 0-6 diary card.
4. Draw approximately 3.5 mL of blood for CBC and differential.
5. Distribute Days 7-13 diary card.

Study Day 134 +/- 2 (14 days after Second Vaccination)

1. Perform basic history and physical exam (including injection site), emphasizing examination of any acute complaints.
2. Record vital signs.
3. Obtain approximately 35 mL of blood for hematology, biochemistry, antibody ELISA and membrane feeding assay.
4. Collect Days 7-13 diary card.
5. Distribute Days 14 – 20 diary card.

Study Day 141 +/- 2 (21 days after second vaccination)

1. Perform basic history and physical exam (including injection site), emphasizing examination of any acute complaints.
2. Record vital signs.
3. Collect Days 14 – 20 diary card.

Study Day 150 +/- 4 (30 days after second vaccination)

1. Perform basic history and physical exam (including injection site), emphasizing examination of any acute complaints.
2. Record vital signs.
3. Obtain approximately 25 mL of blood for antibody ELISA and membrane feeding assay.

Study Day 180 +/- 14 (60 days after second vaccination)

1. Perform basic history and physical exam (including injection site), emphasizing examination of any acute complaints.
2. Record vital signs.
3. Obtain approximately 35 mL of blood for hematology biochemistry, antibody ELISA, and membrane-feeding assay.

Study Day 270 +/- 14 (5 months after second vaccination)

1. Perform basic history and physical exam (including injection site), emphasizing examination of any acute complaints.
2. Record vital signs.
3. Obtain approximately 25 mL of blood for antibody ELISA, and membrane feeding assay.

Study Day 360 +/- 30 (8 months after second Vaccination)

1. Perform basic history and physical exam (including injection site), emphasizing examination of any acute complaints.
2. Record vital signs.
3. Obtain approximately 35 mL of blood for hematology, biochemistry, antibody ELISA and membrane feeding assay.

Study Day 556 +/- 30 (12 months after second Vaccination)

1. Perform basic history and physical exam (including injection site), emphasizing examination of any acute complaints.
2. Record vital signs.
3. Obtain approximately 35 mL of blood for hematology, biochemistry, antibody ELISA and membrane feeding assay.

4. Volunteer is unblinded to vaccination cohort.

Should a volunteer develop a good antibody response, as defined by investigators at the MVDB, that volunteer may be asked if he/she would consider undergoing plasmapheresis. The purpose of plasmapheresis would be to collect sufficient serum to serve as a transmission blocking antibody standard for future trials. The volunteer will be asked to sign a separate consent for the procedure. The volunteer will be under no obligation to undergo plasmapheresis and his/her decision will not affect participation in this or other trials conducted at the Center for Immunization Research..

## Volunteer Symptom Diary

Volunteers will be asked to keep daily symptom diaries recording oral temperature as well as pain/tenderness, redness, swelling at the injection site and any systemic signs or symptoms for 20 days following each immunization (**Appendix E**). The size of any injection-site reaction will be measured using a standardized clear plastic measurement device (**Appendix F**) and recorded in the volunteer symptom diary.

## Laboratory Testing

Using standard techniques, the clinical laboratory will perform the following tests:

1. Complete blood count plus white blood cell differential
2. Serum creatinine
3. AST
4. ALT
5. Blood type and screen (See Section 6.8.2)
6. HIV assay (FDA-approved screening antibody assay with Western Blot confirmation)
7. HBsAg ELISA
8. HCV (FDA-approved screening antibody assay and immunoblot confirmation or viral PCR confirmation)
9. Urinalysis (in the event of an abnormal urine dipstick test)

Urine β-hCG testing will be performed at the clinical trial site using an Food and Drug Administration (FDA)-approved urine pregnancy test kit. Urine dipstick testing will be performed at the trial site using an FDA-approved product.

The anti-Pfs25 and anti-Pvs25 antibody levels and transmission blocking assays for *P. falciparum* will be performed at the MVDB in Rockville, Maryland. The membrane feeding assays for *P. vivax* will be performed at AFRIMS in Thailand. Frozen sera will be shipped from the trial site to the MVDB on dry ice in batches at the discretion of the trial site with consultation from the MVDB. An inventory of each shipment will be maintained by both the trial site and the MVDB.

## Immunologic Testing

### Antibody Assay (ELISA)

Serum antibody levels to the Pfs25 and Pvs25 antigens will be measured by ELISA. Briefly, microwell plates (Dynex Technologies) are coated overnight at 4^o^C with 100 μL/well of antigen solution (1 μg/mL). Plates are washed with TRIS-buffered saline (TBS) containing 0.1% Tween‑20 (0.1% T-TBS) and blocked with TBS containing 5% skim milk powder for 2 hours at room temperature. After washing with 0.1% T-TBS, serum samples at a dilution of 1:5,000 in 0.5% T-TBS containing 5% skim milk powder are added in triplicate and incubated for 2 hours at room temperature. After incubation, unbound antibodies are removed by washing the plates with 0.1% T-TBS, and 100 μL of alkaline phosphatase-conjugated goat anti-human IgG solution (Kirkegaard & Perry Labs, Gaithersburg, Maryland, 1:1,000 dilution in 0.5% T-TBS containing 5% skim milk powder,) is added to each well and incubated for 2 hours at room temperature. Plates are then washed with 0.1% T-TBS, followed by adding 100 μL of phosphatase solution (Sigma, St. Louis, Missouri) to each well; the plates are then covered with aluminum foil and incubated for 20 minutes at room temperature for color development. The plates are read immediately at 405 nm with a microplate reader (Spectramax 340PC Molecular Devices). The optical density values are used to determine anti-Pfs25 and anti-Pvs25 antibody concentration by comparison to a standard curve generated with known positive control sera included on each ELISA plate.

### Transmission Blocking Assay (Membrane Feeds)

Membrane feeds is an ex vivo assay that measures the ability of vaccine-induced antibodies to block the parasite development in mosquito midgut. In this assay, anopheline mosquitoes (*A. stevensi, A. dirus*) were fed blood meals containing malaria–infected erythrocytes and test antiserum through using a membrane feeder. The mosquitoes are subsequently reared and dissected 7-10 days later to determine the number of oocysts present in the midgut. The percent transmission-blocking can then be calculated as 100*(1-test mosquito infection/control mosquito infection). The *P. falciparum*-infected erythrocytes are obtained from the laboratory *P. falciparum* cultures. *P. vivax*-infected erythrocytes are obtained by collecting venous blood from consenting vivax malaria patients (age > 15) who present to Thai Ministry of Public Health clinics. Volunteers’ blood in the PpPfs25/ISA51 & ScPvs25H/ISA51 malaria vaccine trial will be typed and screened at the screening visit to avoid the potential for red cell agglutination while performing the membrane feeding assay.

## Use, Storage, and Tracking of Specimens and Data

Samples and data collected under this protocol will be used to study malaria and related diseases, and possible adverse reactions to vaccination. No genetic testing will be performed. Access to the specimens will be limited using either a locked room or a locked freezer. Samples and data will be stored using codes assigned by the investigators or their designees. Data will be kept in password-protected computers. Only investigators or their designees will have access to the samples and data.

Specimens will be stored at the MVDB in Rockville, Maryland. Specimens will be tracked using Freezerworks, a software database. After completion of the protocol, specimens will be unlinked and used as references in future studies on malaria and related diseases. The research use of stored, unlinked, or unidentified specimens may be exempt from the need for prospective IRB review and approval. Exemption requests will be submitted in writing to the NIH Office of Human Subjects Research, which is authorized to determine whether a research activity is exempt.

Any loss or unanticipated destruction of specimens (for example, due to freezer malfunction) or data (for example, misplacing a printout of data with identifiers) will be reported to the IRBs. Such a loss will be reported to the NIAID IRB as a protocol violation.

Subjects may decide at any point not to have their specimens stored. In this case, the investigator will destroy all remaining identifiable specimens and report to both the subject and to the IRBs on what was done with the specimens. This decision may not affect the subject’s participation in this protocol or any other protocols.

# Adverse Events Monitoring and Reporting

## Definitions

### Adverse Event (AE)

An AE is any untoward medical occurrence in a trial volunteer administered the experimental vaccine and that does not necessarily have a causal relationship with vaccination. An AE can therefore be any unfavorable and unintended sign (including an abnormal laboratory finding), symptom, or disease temporally associated with the use of the investigational vaccine, whether or not related to it. This includes an exacerbation of pre-existing conditions and intercurrent illnesses. Unchanged pre-existing conditions will not be included as an adverse event. All AEs must be graded for intensity and relationship to the investigational vaccine as described in **Sections 7.2.2** and **7.2.3** in this protocol.

### Serious Adverse Event (SAE)

An SAE is an AE, whether considered related to the investigational vaccine or not, meeting one of the following conditions:

1. Death during the period of protocol-defined surveillance
2. Life threatening: defined as an event that places a subject at immediate risk of death at the time of the event and does not refer to an event that hypothetically might have caused death were it more severe
3. Hospitalization during the period of protocol-defined surveillance: defined as at least an overnight stay in the hospital or emergency ward for treatment that would have been inappropriate if administered in the outpatient setting
4. Results in a congenital anomaly or birth defect
5. Results in a persistent or significant disability or incapacity: defined as a substantial disruption of the study volunteer’s ability to carry out normal life functions
6. Any other important medical event that may not result in death, be life threatening, or require hospitalization, may be considered a serious AE when, based upon appropriate medical judgment, the event may jeopardize the subject and may require medical or surgical intervention to prevent one of the outcomes listed above

## Assessment of Adverse Events

### Identification of AEs

Assessment of safety will include clinical observation and monitoring of hematological, chemical and immunologic parameters. Safety will be evaluated by monitoring of volunteers for local and systemic adverse reactions during the course of the trial. Volunteers will be closely observed in the clinic for 30 minutes following each immunization. Additionally, volunteers will return to the clinic on Days 1, 3, 7, 14, 21, 30 and 60 following each vaccination for clinical assessments. In addition they will return to the clinic 9, 12 and 18 months after the first vaccination. For 20 days after each immunization, volunteers will be asked to keep daily diaries of symptoms, recording oral temperature, as well as a subjective assessment of the extent of induration, erythema, pain/swelling at the site of injection, and any systemic signs and/or symptoms. The size of any injection-site reaction will be measured using a standardized clear plastic measurement device and recorded in the volunteer symptom diary. All AEs will be graded for intensity and relationship to study product as described in **Section 7.2.2 and 7.2.3** in this protocol. A study clinician will be available by telephone or pager 24 hours a day during the study evaluation period. Should a volunteer call a study clinician to report an adverse event, it will be determined at that time if an extra visit(s) will be scheduled, and/or appropriate medical advice will be provided. Additionally, all calls will be documented in the volunteer’s study chart, and discussed with the Principal Investigator.

### Determination of Severity

All AEs will be assessed by the investigator using the following protocol-defined grading system:

Grade 0 (None)

Grade 1 (Mild): No effect on activities of daily living

Grade 2 (Moderate): Partial limitation in activities of daily living (can complete ≥ 50% of baseline), or treatment given

Grade 3 (Severe): Activities of daily living limited to < 50% of baseline, or medical evaluation required

Intensity of the following AEs will be assessed by the investigator as described in **Table 3**. All safety laboratory AEs will be graded in severity following the toxicity table in **Appendix G**. Unexpected adverse events not described in the protocol will be graded according to the Cancer Therapy Evaluation Program’s Common Toxicity Criteria, Version 3, National Cancer Institute in **Appendix H**.

| **Table 3: Assessment of Adverse Event Intensity** | | |
| --- | --- | --- |
| **AdverseEvent** | **Grade** | **Intensity** |
| Pain at injection site | 0 | Absent |
|  | 1 | Pain that is easily tolerated |
|  | 2 | Pain that interferes with daily activity |
|  | 3 | Pain that prevents daily activity |
| Erythema at injection site | 0 | 0 mm |
|  | 1 | >0 - <20 mm |
|  | 2 | >20 - <50 mm |
|  | 3 | >50 mm |
| Swelling at injection site | 0 | 0 mm |
|  | 1 | >0 - <20 mm |
|  | 2 | >20 - <50 mm |
|  | 3 | >50 mm |
| Induration at injection site | 0 | 0 mm |
|  | 1 | >0 - <20 mm |
|  | 2 | >20 - <50 mm |
|  | 3 | >50 mm |
| Fever (oral) | 0 | <99.5°F (<37.5°C) |
|  | 1 | >99.5°F - 100.4°F (>37.5°C – 38°C) |
|  | 2 | >100.4°F - 102.2°F (>38°C – 39°C) |
|  | 3 | >102.2°F (>39°C) |
| Headache | 0 | None |
|  | 1 | Headache that is easily tolerated |
|  | 2 | Headache that interferes with daily activity |
|  | 3 | Headache that prevents daily activity |
| Nausea | 0 | None |
|  | 1 | Nausea that is easily tolerated |
|  | 2 | Nausea that interferes with daily activity |
|  | 3 | Nausea that prevents daily activity |
| Malaise | 0 | None |
|  | 1 | Malaise that is easily tolerated |
|  | 2 | Malaise that interferes with daily activity |
|  | 3 | Malaise that prevents daily activity |
| Myalgia | 0 | None |
|  | 1 | Myalgia that is easily tolerated |
|  | 2 | Myalgia that interferes with daily activity |
|  | 3 | Myalgia that prevents daily activity |
| Arthralgia | 0 | None |
|  | 1 | Joint pain that is easily tolerated |
|  | 2 | Joint pain that interferes with daily activity |
|  | 3 | Joint pain that prevents daily activity |
| Urticaria | 0 | None |
|  | 1 | Requiring no medications |
|  | 2 | Requiring PO or topical treatment or IV medication or steroids for <24 hours |
|  | 3 | Requiring IV medication or steroids for >24 hours |

### Association with Receipt of the Study Vaccine

All AEs will have their possible relationship to study vaccine assessed using the following terms:

Definite: Clear-cut temporal association, and no other possible cause.

Probable: Clear-cut temporal association and a potential alternative etiology is not apparent.

Possible: Less clear temporal association; other etiologies also possible.

Remote: Temporal association between the AE and the vaccine or the nature of the event is such that the vaccine is not likely to have had any reasonable association with the observed illness/event (cause and effect relationship improbable but not impossible).

Not Related: The AE is completely independent of vaccine administration; and/or evidence exists that the event is definitely related to another etiology.

The degree of certainty with which an AE can be attributed to administration of the study vaccine will be determined by how well the event can be understood in terms of one or more of the following:

1. The event being temporally related with vaccination or reproduced on re-vaccination.
2. A reaction of similar nature having previously been observed with this type of vaccine and/or formulation.
3. The event having often been reported in the literature for similar types of vaccines.

All local (injection-site) reactions will be considered causally related to vaccination.

## Adverse Event Reporting

All SAEs will be reviewed by a study physician, recorded on the appropriate SAE form, and followed through to resolution by a study physician. All SAEs will be reported by telephone or fax within 1 working day of notification of the SAE occurrence to all of the following:

- IND Sponsor, Regulatory Compliance and Human Subjects Protection Branch Safety Section (RCHSPB Safety)/NIAID: Phone: 301-846-5301, Fax: 301-846-6224; email: RCHSPSafety@mail.nih.gov.
- Committee on Human Research (Johns Hopkins IRB): Phone: 1-888-262-3242, Fax: 410-955-0258
- NIAID IRB: Phone: 301-435-9273, Fax: 301-480-6606

Following notification from the investigator, RCHSPB as the IND sponsor, will report events that are both serious and unexpected that are possibly, probably or definitely related to the vaccine, to the FDA within the required timelines: fatal and life-threatening events within 7 calendar days (by phone or fax) and all other SAEs in writing within 15 calendar days. All SAEs not listed as possibly, probably or definitely related will be reported to the FDA at least annually in a summary format and to the IRBs as required by the institution. All local and systemic reactions not meeting the criteria for “serious adverse events” will be captured on the appropriate case report form (CRF). These events will be followed to resolution.

## Adverse Event Monitoring

### Medical Monitor

An independent Medical Monitor has been appointed for oversight of safety in this trial. The Medical Monitor will be available to advise the investigators on trial-related medical questions or problems, and act as a representative for the volunteers’ welfare. Additionally, the Medical Monitor may ask to convene a safety monitoring committee meeting for review of any safety issue or adverse event. The Medical Monitor will review any SAE determined to be possibly, probably, or definitely related to vaccination.

### Safety Monitoring Committee

MVDB, in consultation with RCHSPB, will select three independent monitors to advise RCHSPB and the study investigators on the trial. These individuals will be independent of MVDB and the clinical trial site. The SMC’s primary responsibility will be to monitor volunteer safety. The Principal Investigator is responsible for ensuring that the SMC is aware of all new safety information. The SMC will periodically review individual and cumulative volunteer data on safety and enrollment when making recommendations regarding the safe continuation of the study. If no stopping criteria are met (**Section 7.5**), dose escalation will proceed with approval from the SMC. The SMC will review cumulative safety data for evidence of study-related AEs, adherence to the protocol, and factors that may affect outcome or study data such as protocol violations and losses to follow-up. Because of the staggered start for each dose group, the SMC will meet after the second part of the dose group (n=8) has been evaluated for at least 14 days post-vaccination; such that 35 days of safety data will have accumulated for the first 4 vaccinees, and 21 days of safety data will have accumulated for the remaining 8 vaccinees. The SMC will review the cumulative safety data following the first vaccination of the lower dose group to approve dose escalation, as well as following the second vaccination prior to dose escalation, and whenever the need should arise, to review study conduct and cumulative safety data, as well as at the discretion of the SMC and/or study investigators.

## Stopping Criteria

If a dose of vaccine is considered unacceptably reactogenic, dose escalation and/or additional vaccinations will be suspended until reviewed by the SMC and IND sponsor (RCHSPB). The communications from the SMC will subsequently be forwarded by the investigators to the NIAID IRB and the clinical trial site IRB. All local adverse events will be routinely reviewed by the SMC prior to granting approval for dose escalation.

The following criteria will be used to define unacceptable reactogenicity of either of the malaria vaccines. PpPfs25H and ScPvs25H will be considered separately:

1. One or more volunteers experience a SAE (as defined in **Section 7.1.2** in this protocol) that is determined to be possibly, probably or definitely related to the vaccine, **OR**
2. One or more volunteers experience hypersensitivity reaction (a Grade 3 or 4 allergic reaction, as defined **Appendix H**) that is probably or definitely related to the vaccine, **OR**
3. Any severe clinical illness occurs that is not explained by a diagnosis that is unrelated to vaccination, **OR**
4. Three or more volunteers in a single dose cohort experience Grade 2 or higher injection site induration or abscess formation requiring drainage for diagnosis and treatment. A volunteer who experiences Grade 2 or higher injection site induration/abscess following the first vaccination, may not receive the final vaccination, **OR**
5. One or more volunteers in a single-dose cohort experience a Grade 3 or higher safety laboratory abnormality (see **Appendix G**), or Grade 3 systemic AE that is determined to be possibly, probably or definitely related to the vaccine, as defined in **Section 7.2.2** in this protocol.

# Data Collection and Monitoring

## Source Documentation

Complete source documentation (laboratory test reports, hospital or medical records, etc.) is required for every study subject for the entire duration of the study. Case Report Forms (CRFs) and volunteer symptom diaries will be used to record data for subjects enrolled in the study. The Investigator is responsible for the accuracy and completeness of the data reported to the IND Sponsor in the CRFs and diaries. Data reported in the CRFs derived from source documents should be consistent with source documents or the discrepancies should be explained.

## Study Documentation

Study-related documentation will be completed as required by the IRBs, the IND Sponsor and regulatory authorities. Continuing review documentation will be submitted by the Investigator to the IRBs on the anniversary date of initial review as specified by each IRB. An annual report will be submitted by the RCHSPB to the FDA on the anniversary date that the IND went into effect. These reports will provide a brief description of the progress of the investigation as outlined in 21 *Code of Federal Regulations* 312.33 and will include any revisions of the protocol.

The Investigator will maintain adequate records of the disposition of the investigational product, including dates, quantity and use by subjects. If the study is terminated, suspended or completed, final disposition of unused vaccine supplies will be determined by the Malaria Vaccine Development Branch, in conjunction with the IND Sponsor.

In addition to the study-related documentation required by the regulatory authorities, the MVDB will also submit two reports to the IND Sponsor. The first, or interim, report will be completed after the serologic data from the 5 month (20 weeks) blood draw has been compiled. This interim report, based on the serologic response that is observed 1 month after the second immunization, will serve as the basis for deciding whether to continue with future Phase 1 testing of the formulation. A final report will be submitted by the MVDB to the IND Sponsor after trial completion.

## Retention of Records

Trial-related documents will be maintained by the Investigator for a period of 2 years after final marketing approval of the vaccine, or for 2 years following the formal discontinuation of clinical development of the product. The IND Sponsor is required to inform the Investigator as to when such documents need no longer be retained. Storage of all trial-related documents will be such that confidentiality will be strictly maintained.

## Protocol Revisions

No revisions to this protocol will be permitted without documented approval from both the IND Sponsor and the IRBs that granted the original approval for the study. This does not apply to changes made to reduce discomfort or avert risk to study volunteers. Furthermore, in the event of a medical emergency, the Investigator shall perform any medical procedures that are deemed medically appropriate. The Investigator must notify the IND Sponsor of all such occurrences. Any change to the protocol will be submitted to the participating IRBs as a protocol amendment and changes not affecting risk to volunteers may be expedited, as appropriate.

## Clinical Investigator’s Brochure

Investigators will receive the current version of the Clinical Investigator’s Brochure, which comprehensively describes all the available preclinical experience with the experimental vaccine. If relevant new information becomes available during the course of the trial, the Investigators will receive a revised Investigator’s Brochure or an amendment to the current version.

## Study Monitoring

The IND Sponsor will monitor through delegated responsibility, all aspects of the study, with respect to current Good Clinical Practices (GCP), for compliance with applicable government regulations. Prior to the start of the study, the Investigator will be informed of the frequency of monitoring visits and will be given reasonable notification prior to each visit. The objectives of a monitoring visit will be to verify the prompt reporting of SAEs, to check the availability of the signed Informed Consent, GCP adherence to the protocol, and to compare CRFs and spreadsheets with source documents for completeness and accuracy. During the monitoring visit, the Investigator (and/or designee) and other study personnel should be available to discuss the study. Study documents must be available for review throughout the course of the study. The IND Sponsor will retain originals of the FDA Form 1572 and copies of other study documents as deemed necessary.

# Statistical Considerations

## General Design

The goal of this Phase 1 vaccine trial is to demonstrate safety, reactogenicity, and immunogenicity of the PpPfs25/ISA51 and ScPvs25/ISA51 malaria vaccines in human volunteers. The results from this trial will be used to determine dosing and scheduling in subsequent trials.

### Description of the Statistical Methods to Be Employed

The purpose of this trial is to estimate event rates and patterns of immune responses as well as to compare these rates and patterns in different doses of the study vaccines and to compare the response by the two vaccines. This section briefly describes the statistical methods to be used. A detailed analytic plan will fully describe the methods. The analytic plan will discuss the planned approaches to missing data. Listings will show all observed data and, if applicable, impute values and the approaches taken for imputation.

Estimates will be presented with their 95% confidence intervals. Descriptive approaches will be used to meet the protocol objectives as stated in **Section** **2.0** of this protocol. In particular, the immunologic response for each of the 72 volunteers will be presented as individual graphs,

and all adverse events will be listed. Formal statistical tests, as outlined below, will be used to compare doses.

Statistical tests on the adverse event data will be performed without correction for multiplicity, because typically the family-wise Type I error is not bound for tests of safety. A nominal Type I error rate of 10 percent will be used. Most of the analyses of immunogenicity will be based on a longitudinal mixed model with terms for dose group for each of the 2 vaccines.

Primary Objective: To assess the safety, reactogenicity, and immunogenicity of the PpPfs25/ISA51 and ScPvs25/ISA51 malaria vaccines.

1. Summarize the frequency of immediate, systemic and local AEs.
2. Line listing of individual clinical and laboratory AEs as classified by immediate (within the first 30 minutes), systemic and local will be displayed in tabular format and stratified by dose cohort.
3. AEs will be summarized by severity and relationship to vaccine by individuals and dose cohort.
4. The proportion of volunteers with at least one local adverse event will be compared by dose cohort for each vaccine.  Formal statistical tests will assess whether the three cohorts differ with respect to these proportions, and whether there is a dose-response relationship. To see if there is a difference in adverse events between the initial vaccination and the subsequent vaccination we perform a sign test, where the response for each volunteer is the difference between the number of adverse events in the 60 days following the first vaccination and the number of adverse events in the 60 days following the second vaccination.
5. To determine the dose that generates the highest serum antibody levels to homologous antigen at Day 150 (1 month after the second immunization).

 i.   A Kruskal-Wallis test will be performed to check for any significant differences in the antibody concentrations of the three dose groups.  If that test is significant, then all three pairwise Mann-Whitney tests will be performed to see which pairs have significant differences. If the Kruskal-Wallis test is not significant then we will not perform any Mann-Whitney tests on the pairs of dose groups.  All of the tests can be performed at the 0.05 level and they will retain a family-wise error rate of 0.05

Secondary Objective 1: To assess and compare the duration of specific antibody response to homologous Pfs25 or Pvs25 over an 18 month period.

1. Describe immunogenicity responses by vaccine and dose group, over time.
2. Individual responses will be described over time and stratified by dose cohort.
3. Antibody concentration will be measured at Days 0, 14, 30, 60, 90, 120, 134, 150, 180, 270, 360 and 556. To exploit the multiple measures of antibody within each subject, a longitudinal model will be built to describe the antibody response over time. The model will explore if there are any differences between Pfs25 and Pvs25 and explore dose effects.

Secondary Objective 2: Measure the effect of boosting at 4 months on antibody units.

1. Using difference in antibody level from Day 0 to Day 150 as the response, exact Jonckheere-Terpstra tests will be performed to determine if there is a dose response. Using the same response, exact stratified Wilcoxon rank sum tests, stratified on dose level, will be performed to determine if there are differences between the dose groups. If feasible, similar tests may be done using the longitudinal model described above.
2. In addition, the increase in the levels of antibody between Day 120 and Day 150 will be estimated and compared.
3. Waning will be assessed by comparing levels of antibody at Days 180, 270, 360 and 556 with the level at Day 150.

The following tertiary objectives are for information only:

Tertiary Objective 1: To measure the ability of the vaccine induced antibody to significantly reduce or block oocyst development in the mosquito as measured by the membrane feeding assay for both Pvs25 and Pfs25.

1. Graphs will display growth inhibition (the ability of the antibody to significantly reduce or block oocyst development) expressed as a percent of inhibition comparing test sera to pre-immune sera.
2. Depending on the distribution of the data, parametric or non-parametric methods will be used to compare inhibition as a function of dose, and serotype.

Tertiary Objective 2: To determine the relationship between antibody levels and degree of in vitro oocyst development.

Non-linear regression will be used to model growth inhibition and the ELISA value giving 50% growth inhibition will be estimated.

Should the need arise for terminating the study early, the investigative team will discuss with the SMC the reason for termination and determine which study questions can be addressed in an unbiased manner with the available data. The available data will be analyzed and interpreted in light of early termination. Deviations from the statistical plan will be reported in the study report.

### Safety

The primary safety endpoint is the frequency and severity of vaccine-related AEs, as classified by both intensity and severity through active and passive surveillance. Separate assessments of systemic and local reactions will be performed.

### Immunogenicity Analysis

The primary immunogenicity endpoint will be evaluation of day 150 sera (1 month after second vaccination). Antibodies will be measured by ELISA on Days 0, 14, 30, 60, 90, 120, 134, 150, 180, 270, 360, and 556 as listed in the schedule of visits (**Appendix D**). Additionally, the membrane feeding assay will be performed on Days 0, 60, 120, 134, 150, 180, 270, 360 and 556 as a way to assess the biologic activity of the vaccines.

## Sample Size

Based on an analysis of the human antibody responses to a number of malaria antigens that have been tested in clinical trials (the three components of Combination B [6] and RTS,S [7]), the observed coefficient of variation in the range of antibody concentrations has been found to be remarkably constant at approximately 1.2 - 1.4. Based on the distribution of antibody responses for each of the antigens in the combination B and RTS,S trials, a sample size of 10 volunteers per dose group would permit detection of at least a five-fold difference in antibody concentration between groups using a Mann-Whitney test, assuming a level of significance of 0.05 and a power of 0.80. Additionally, a group size of 10 volunteers per dose gives 0.80 probability for detecting one or more serious or severe AE that occurred with a probability of 0.15 per volunteer.

# Protection of Human Subjects

## Institutional Review Board/Ethics Committee

The Investigator will be responsible for obtaining IRB approval for the study. Before the start of the study, the appropriate documents (including the Protocol, Investigator’s Brochure, Informed Consent Form, information sheets, CRFs and advertisements) will be submitted to the IRB. A copy of the study approval (including approval of the informed consent form) is to be maintained in the Investigator’s study document binder and a copy will be supplied to the IND Sponsor. During the study, the Investigator is responsible for providing the IRB with all documents subject to review (i.e., Protocol Amendments, informed consent form updates, advertisements, and any written information that may be provided to the subject). Annual reports on the progress of the study will be made to the IRBs by the Investigator in accordance with IRB guidelines and government regulations.

## Informed Consent

In obtaining and documenting informed consent, the Investigator must comply with the applicable regulatory requirements, Good Clinical Practices and ethical principles. The written informed consent form must be approved by all IRBs prior to its use.

## Risks

Risks to the volunteers are associated with venipuncture and with immunization. These risks are outlined below.

Female volunteers will be cautioned of the unknown risk of study vaccines to the fetus and will be advised to use adequate birth control methods for the duration of the study.

### Venipuncture

Risks occasionally associated with venipuncture include pain and bruising at the site of venipuncture, infection, lightheadedness, and syncope (rarely).

### Immunization

Possible local vaccine reactions include pain, swelling, erythema, induration, abscess formation, limitation of limb movement for several days, lymphadenopathy or pruritus at the injection site. Severe swelling and/or induration at the injection site have been observed in some volunteers who received PpPfs25/ISA51 or ScPvs25/ISA51. Systemic reactions such as fever, chills, headache, fatigue, malaise, myalgia and joint pain may also possibly occur. A leukemoid reaction was noted in one volunteer two weeks after receipt of the second 5 μg dose of PpPfs25/ISA51. Immediate hypersensitivity reactions including urticaria, anaphylaxis or other IgE mediated responses are possible as with any vaccine. As with any investigational vaccine, there is a theoretical possibility of risks about which we have no present knowledge. Volunteers will be informed of any such risks should further data become available.

## Benefits

Volunteers will not receive any direct benefit from participation in this study. It is hoped that information gained in this study will contribute to the development of a safe and effective malaria vaccine.

## Confidentiality

All study-related information will be stored securely at the study site. All volunteer information will be stored in locked file cabinets in areas with access limited to study staff. All laboratory specimens, reports, study data collection, process and administrative forms will be identified by coded number only to maintain volunteer confidentiality. All computer entry will be done by coded number only, and all local databases will be secured with password-protected access systems. Forms, lists, logbooks, appointment books and any other listings that link volunteer ID numbers to other identifying information will be stored in a separate, locked file in an area with limited access.

Volunteers’ study information will not be released without the written permission of the volunteer, except as necessary for monitoring by NIAID and/or its contractors and the FDA.

## Compensation

Volunteers will be paid $30.00 for their screening visit and $25.00 per visit during participation in the study ($530.00 for 21 visits) for their time and inconvenience. Volunteers will be paid for the screening visit, only if enrolled. A bonus of $200.00 will be paid for completion of all visits. The total payment will be divided over the course of the study with the bonus dispensed upon completion of the trial.

# References

World Health Organization, Tropical Disease Research. Source: World Health Report 2002. <http://www.who.int/tdr/diseases/malaria/diseaseinfo.htm>

Mendix K, Sina BJ, Marchesini P, and Carter R. The neglected burden of *P. vivax* malaria. *Am J Trop Med Hyg*, Jan-Feb;64(1-2 supple):97-106.

Gringeri A, Santagostino E, Muca-Perja M, et al. Safety and immunogenicity of HIV-1 Tat toxoid in immunocompromised HIV-1-infected patients. J Hum Virol 1998;1(4):293-8.

4. Carr A, Rodriguez E, Arango Mdel C, et al. Immunotherapy of advanced breast cancer with a heterophilic ganglioside (NeuGcGM3) cancer vaccine. J Clin Oncol 2003;21(6):1015-21.

5. Limsuwan A, Churdboonchart V, Moss RB, et al. Safety and immunogenicity of REMUNE in HIV-infected Thai subjects. Vaccine 1998;16(2-3):142-9.

1. Saul, A., Lawrence G., Smillie A., et al. Human Phase 1 vaccine trials of 3 recombinant asexual stage malaria antigens with Montanide® ISA 720 adjuvant. *Vaccine* 17:3145-3159 (1999).
2. Stoute, J.A., Slaoui, M., Heppner, D.G., et al. A preliminary evaluation of a recombinant circumsporozoite protein vaccine against Plasmodium falciparum malaria. RTS,S Malaria Vaccine Evaluation Group. *N Engl J Med* 336:86-91 (1997).

## Appendix A – Volunteer Recruitment Advertisements

| **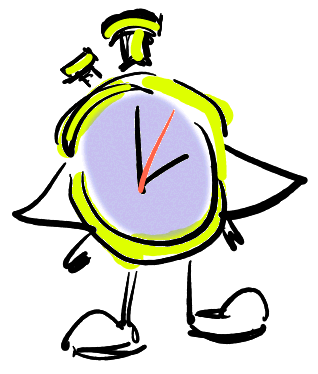Every Minute ~**  **1,000 people worldwide**  **get infected with dangerous,**  **mosquito-borne diseases.**  **Many Will Die.**  **Healthy Volunteers needed for**  **Investigational Malaria Vaccine Studies**  **Near Foggy Bottom/GWU Metro Stop.**  **You’ll be compensated for your time.**  **Call Toll free: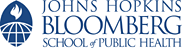 1-877-829-4968** |
| --- |

**Center for Immunization Research**

***Principal Investigator:Anna Durbin,MD***

| **There are places in the world where**  **Widespread Mosquito Diseases**  **kill1,500,000 people each year*!***  **Help us find a solution*!***  **Healthy Volunteers needed for**  **Investigational Malaria Vaccine Studies**  **Near Foggy Bottom/GWU Metro Stop**  **You’ll be compensated for your time.**  **Call Toll Free: 1-877-829-4968** 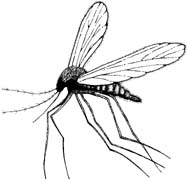 **Widespread**  **Mosquito**  **Diseases**  **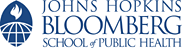** |
| --- |

**Center for Immunization Research**

***Principal Investigator:Anna Durbin,MD***


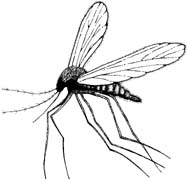


**Widespread**

**Mosquito**

**Diseases**

# Appendix B – Volunteer Phone Screen

# Appendix C – Informed Consent Comprehension Exam

# Johns Hopkins University

# Center for Immunization Research

____________

Screen #

**Malaria**

____________

# Vol # Comprehension Exam

1. As part of this study, you’ll be injected with a live malaria parasite... **T F**

2. This vaccine will protect you from getting malaria. **T F**

3. There is a chance you could have a reaction to this vaccine, such as

an allergic reaction, or redness, or swelling of your arm. **T F**

4. Women enrolled in this study must use birth control for the duration

of the study. **T F**

5. If you change your mind about being in the study after you are vaccinated,

you can withdraw your consent. **T F**

6. It’s OK to enroll in other investigational agent studies when you are still

in this study. **T F** **T F**

7. This vaccine has been given to hundreds of people already,

so we know it is completely safe. **T F**

8. You’ll have to fill out a diary card for 3 weeks after each vaccination

and bring it to the clinic. **T F**

9. You’ll have your blood drawn as part of this study. **T F**

10. You’ll get 2 vaccinations (shots) in this study. **T F**

11. If you feel sick during the study, you should keep it to yourself. **T F** **T F**

12. It’s OK to be in this study if you have been in other malaria studies in the past. **T F** **T F**

13. If you join the study, you need to be followed in our clinic for 18 months. **T F** **T F**

14. Before joining the study, you’ll be tested for HIV, hepatitis B, & hepatitis C. **T F** **T F**

15. Everybody in this study will get the same dose of vaccine. **T F**

Total number correct before review: ______

Total number correct after review: ______

Reviewed by Date ____/____/____

# Appendix D – Schedule of Visits

| Procedures | Blood Volume | Month |  | 0 |  |  |  |  |  | 1 | 2 | 3 | 4 |  |  |  |  |  | 5 | 6 | 9 | 12 | 18 |
| --- | --- | --- | --- | --- | --- | --- | --- | --- | --- | --- | --- | --- | --- | --- | --- | --- | --- | --- | --- | --- | --- | --- | --- |
|  |  | Day | Screen | 0 | 1 | 3 | 7 | 14 | 21 | 30 | 60 | 90 | 120 | 121 | 123 | 127 | 134 | 141 | 150 | 180 | 270 | 360 | 556 |
| Complete History and Physical |  |  | X |  |  |  |  |  |  |  |  |  |  |  |  |  |  |  |  |  |  |  |  |
| Obtain Informed Consent |  |  | X |  |  |  |  |  |  |  |  |  |  |  |  |  |  |  |  |  |  |  |  |
| Interim Clinical Evaluation |  |  |  | X | X | X | X | X | X | X | X | X | X | X | X | X | X | X | X | X | X | X | X |
| Distribute diary card |  |  |  | X |  |  | X | X |  |  |  |  | X |  |  | X | X |  |  |  |  |  |  |
| Collect diary card |  |  |  |  |  |  | X | X | X |  |  |  |  |  |  | X | X | X |  |  |  |  |  |
|  |  |  |  |  |  |  |  |  |  |  |  |  |  |  |  |  |  |  |  |  |  |  |  |
| CBC & Differential | 5 mL |  | X | X |  | X |  | X |  |  | X |  | X |  | X |  | X |  |  | X |  | X | X |
| AST/ALT & Creatinine | 5 mL |  | X | X |  | X |  | X |  |  | X |  | X |  | X |  | X |  |  | X |  | X | X |
| Type & Screen | 5 mL |  | X |  |  |  |  |  |  |  |  |  |  |  |  |  |  |  |  |  |  |  |  |
| Urinalysis |  |  | X |  |  |  |  |  |  |  |  |  |  |  |  |  |  |  |  |  |  |  |  |
| Urine pregnancy test (females) |  |  | X | X |  |  |  |  |  |  |  |  | X |  |  |  |  |  |  |  |  |  |  |
| HIV  HCV  HBsAg | 10 mL |  | X |  |  |  |  |  |  |  |  |  |  |  |  |  |  |  |  |  |  |  |  |
|  |  |  |  |  |  |  |  |  |  |  |  |  |  |  |  |  |  |  |  |  |  |  |  |
| VACCINATION |  |  |  | X |  |  |  |  |  |  |  |  | X |  |  |  |  |  |  |  |  |  |  |
| Anti-Pxs25 antibody ELISA | 5 mL |  |  | X |  |  |  | X |  | X | X | X | X |  |  |  | X |  | X | X | X | X | X |
| Membrane Feeding Assay | 20 mL |  |  | X |  |  |  |  |  |  | X |  | X |  |  |  | X |  | X | X | X | X | X |
|  |  |  |  |  |  |  |  |  |  |  |  |  |  |  |  |  |  |  |  |  |  |  |  |
| Blood Volume (mL) |  |  | 25 | 35 |  | 10 |  | 15 |  | 5 | 35 | 5 | 35 |  | 10 |  | 35 |  | 25 | 35 | 25 | 35 | 35 |
| Cumulative Blood Volume (mL) |  |  | 25 | 60 |  | 70 |  | 85 |  | 90 | 125 | 130 | 165 |  | 175 |  | 210 |  | 235 | 270 | 295 | 330 | 365 |

# Appendix E – Volunteer Diary Cards

**((INSIDE of Day 0-6 card)**

## Appendix F – Injection Site Reaction Measuring Tool


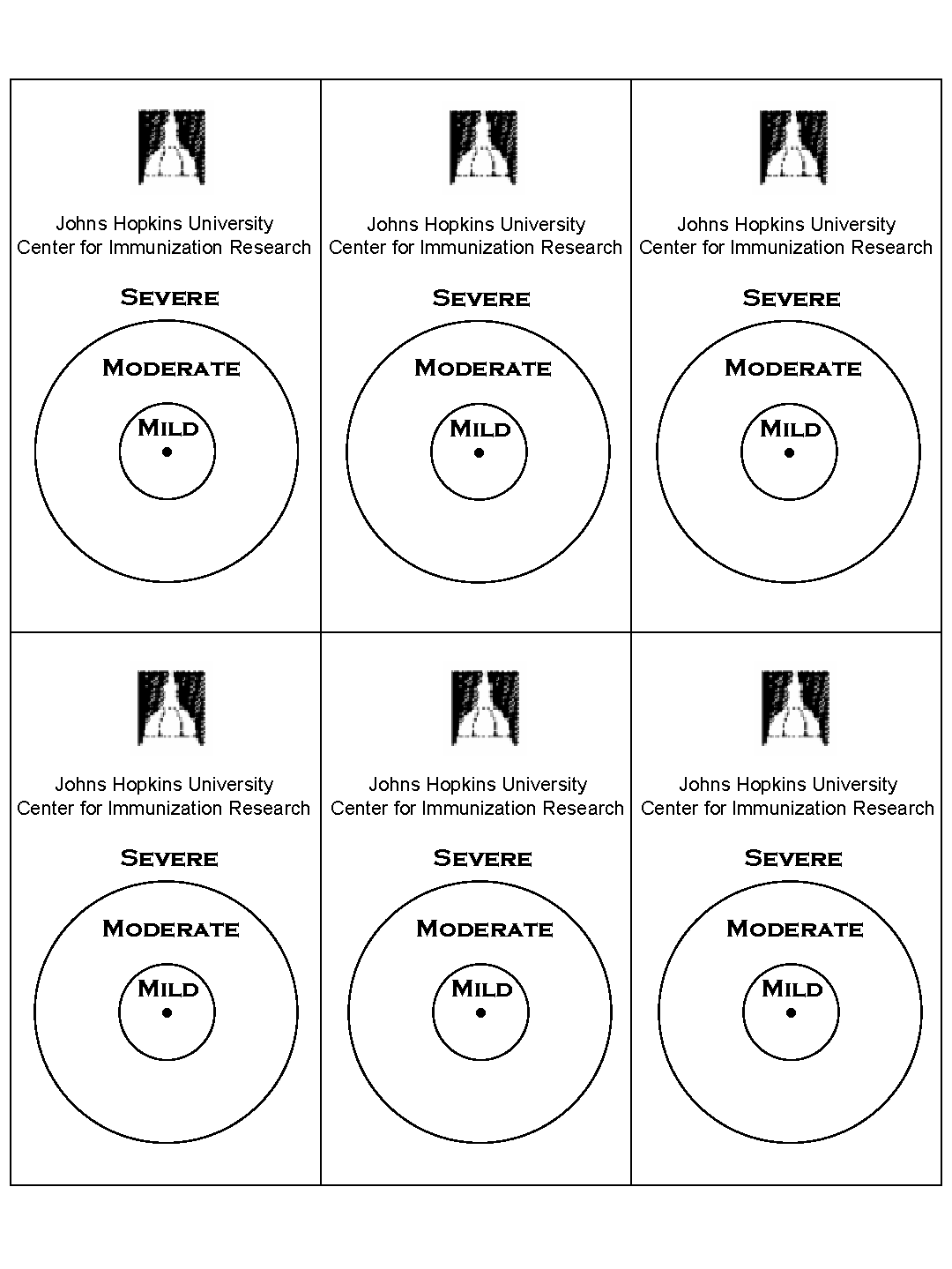


## Appendix G – Toxicity Table for Grading Laboratory Adverse Events

These tables are to be used to assess laboratory adverse events for those tests to be performed as part of the PpPfs25/ISA51 and ScPvs25/ISA51 malaria vaccines clinical trial protocol.

**ABBREVIATIONS:** Abbreviations utilized in the Table:

ULN = Upper Limit of Normal

LLN = Lower Limit of Normal

**ESTIMATING SEVERITY GRADE**

**GRADE 1 Mild:** no effect on activities of daily living; no medical intervention/therapy required

**GRADE 2 Moderate:** partial limitation in activities of daily living (can complete > 50% of baseline); no or minimal medical intervention/therapy required

**GRADE 3 Severe:** activities of daily living limited to < 50% of baseline; medical evaluation/therapy required

| **Laboratory** | **Mild (Grade 1)** | **Moderate (Grade 2)** | **Severe (Grade 3)** |
| --- | --- | --- | --- |
| Hgb (♀) – change from baseline value in gm/dl | ≥ 1.0 & < 1.5 | ≥ 1.5 & < 2.0 | ≥ 2.0 |
| Hgb (♂) – change from baseline value in gm/dl | ≥ 1.5 & < 2.0 | ≥ 2.0 & < 2.5 | ≥ 2.5 |
| WBC – cells/mm^3^  (Increase in WBC) | ≥ 11000 & < 15000 | ≥ 15000 &  < 20000 | ≥ 20000 |
| WBC – cells/mm^3^  (Decrease in WBC) | < 3500 & ≥ 2500 | < 2500 &  ≥ 1500 | < 1500 |
| Platelets – cell/mm^3^ | < 135,000 & ≥ 125,000 | < 125,000 & ≥ 100,000 | < 100,000 |
| AST/ALT (increase by factor) | > 1.0 & < 2.5 x ULN | ≥ 2.5 & < 4 x ULN | ≥ 4 x ULN |
| Serum creatinine – mg/dL | IN* - IN+0.2 | > IN+0.2 - < 2.0 | ≥ 2.0 |

| **URINALYSIS** | | | |
| --- | --- | --- | --- |
|  | Grade 1 | Grade 2 | Grade 3 |
| Proteinuria | 2+  or  0.5 - 1 gm loss/day | 3+  or  1 - 2 gm loss/day | 4+  or  2 - 3.5 gm loss/day |
| Hematuria | 5-10 rbc/hpf | >10 rbc/hpf | gross, with or without clots, OR red blood cell casts |

## Appendix H – Common Toxicity Criteria for Grading Unexpected Adverse Events
